# *Zanthoxylum bungeanum* Maxim. (Rutaceae): A Systematic Review of Its Traditional Uses, Botany, Phytochemistry, Pharmacology, Pharmacokinetics, and Toxicology

**DOI:** 10.3390/ijms18102172

**Published:** 2017-10-18

**Authors:** Mengmeng Zhang, Jiaolong Wang, Lei Zhu, Tao Li, Weidong Jiang, Juan Zhou, Wei Peng, Chunjie Wu

**Affiliations:** 1 College of Pharmacy, Chengdu University of Traditional Chinese Medicine, Chengdu 611137, China; garita119@163.com (M.Z.); helvegps4@126.com (J.W.); zld163wyyx@163.com (L.Z.); litcdtcm@126.com (T.L.); 2 Sichuan Institute for Food and Drug Control, Chengdu 611731, China; jwd-322@163.com (W.J.); zhoujuan009@163.com (J.Z.)

**Keywords:** *Zanthoxylum bungeanum* Maxim., traditional usages, botany, phytochemistry, pharmacology, pharmacokinetics, toxicology

## Abstract

*Zanthoxylum bungeanum* Maxim. (Rutaceae) is a popular food additive and traditional Chinese herbal medicine commonly named *HuaJiao* in China. This plant is widely distributed in Asian countries. The aim of this paper is to provide a systematic review on the traditional usages, botany, phytochemistry, pharmacology, pharmacokinetics, and toxicology of this plant. Furthermore, the possible development and perspectives for future research on this plant are also discussed. To date, over 140 compounds have been isolated and identified from *Z. bungeanum*, including alkaloids, terpenoids, flavonoids, and free fatty acids. The extracts and compounds have been shown to possess wide-ranging biological activity, such as anti-inflammatory and analgesic effects, antioxidant and anti-tumor effects, antibacterial and antifungal effects, as well as regulatory effects on the gastrointestinal system and nervous system, and other effects. As a traditional herbal medicine, *Z. bungeanum* has been widely used to treat many diseases, especially digestive disorders, toothache, stomach ache, and diarrhea. Many traditional usages of this plant have been validated by present investigations. However, further research elucidating the structure-function relationship among chemical compounds, understanding the mechanism of unique sensation, as well as exploring new clinical effects and establishing criteria for quality control for *Z. bungeanum* should be further studied.

## 1. Introduction

The *Zanthoxylum* genus (Rutaceae) consists of 250 species worldwide, including 45 species and 13 varieties in China. *Z. bungeanum* is a species of the genus *Zanthoxylum*, widely distributed in Asian countries including China, Japan, India, Korea, etc. The fruits of *Z. bungeanum* are the most popular commercial product in the genus *Zanthoxylum*, and are largely used as a popular condiment in cooking and medicine with a long history for both medicinal and economic uses in China. So far, multiple cultivars of *HuaJiao* have been cultivated during the process, such as *Yuexigong Jiao*, *Da Hongpao*, and *Hanyuan HuaJiao* [1,2]. The pericarps color of *Z. bungeanum* cultivars is bright red, and therefore these cultivars are commonly known as “*HonghuaJiao*” in Chinese [3,4].

Since 1977, the *Z. bungeanum* has been listed in the Pharmacopoeia of the People’s Republic of China (Ch. P), and over 30 prescriptions containing *Z. bungeanum* have been applied for the treatment of abdominal pain, toothache, dyspepsia, vomiting, diarrhea, ascariasis, eczema, etc. [5,6,7]. With increasing research on *Z. bungeanum*, more than 140 chemical compounds have been isolated and identified from this plant, which includes alkaloids, terpenoids, flavonoids, and free fatty acids, as well as a small amount of inorganic elements [8,9,10,11,12]. In addition, recent investigations have revealed that a wide range of pharmacological activities have been found in *Z. bungeanum*, including analgesic effects, anti-inflammatory effects, antifungal and antibacterial effects, antioxidant and anti-tumor effects, effects on the digestive and circulatory system, as well as other effects. Currently, *Z. bungeanum* pericarps remain an important traditional Chinese medicine listed in the Ch.P, and their essential oils are regarded as the indicator agent for evaluating the quality of *Z. bungeanum* pericarps [6,7].

In this article, the relevant literature on *Z. bungeanum* was collected from Chinese medicine books, a series of articles of PhD. and MSc. Dissertations and scientific databases, including PubMed, Sciencedirect, Web of Science, Springer, Baidu Scholar, Elsevier, Wiley, CNKI, and others. We systematically review the multi-faceted literature on *Z. bungeanum*, including the traditional usages, the botany, and the advance of phytochemistry, pharmacology, pharmacokinetics, as well as the toxicology of this plant. Furthermore, possible research directions and new perspectives on *Z. bungeanum* are also discussed.

## 2. Traditional Usages

*Z. bungeanum* cultivation has a long history, dating back over 2000 years both for its medicinal and economic value in China. The first description of this plant can be traced back to Shijing 2000 years ago, recording that *Z. bungeanum* was always regarded as a precious token due to its fruitful and bright red fruits. As early as the period of the pre-Qin Dynasty, the pericarps of *Z. bungeanum* have commonly been used as a special spice in important activities, with this being the earliest utilization of *Z. bungeanum* in folk life [13]. In addition, pericarps are the main part of *Z. bungeanum* used as a Chinese medicine, and they are commonly stir-fried or processed by vinegar and salt-water [14]. The pharmacological effects of this plant were first listed in *ShenNong BenCaoJing* (the earliest Traditional Chinese Medicine (TCM) monograph during the Eastern Han Dynasty); it was used as an herbal medicine with the function of strengthening teeth, improving eyesight, and removing cold-dampness [15]. Then, the subsequent monograph, *Mingyi Bielu*, described *Z. bungeanum* with good blood-promoting activity, joint-smoothing properties, meridians-regulating and teeth-strengthening properties. In *Zhenglei Bencao*, another famous monograph on traditional Chinese medicine, *Z. bungeanum* was described as a treatment for throat impediment, vomiting, and postpartum abdominal pain, and in the Compendium of Materia Medica (*Bencao Gangmu*), *Z. bungeanum* pericarps were described as a good treatment for toothache, diarrhea, ascariasis, swelling, dampness, and others [15,16,17]. In addition, *Z. bungeanum* was also recorded in other classic monographs of medicine including *Yaoxinglun*, *Shiliao bencao*, *Bencao xinbian*, *Bencao tujing* and others. The pericarps are attributive to the heart and spleen meridians with the properties of being pungent in taste and warm in nature. *Z. bungeanum* has a warming action, thus relieving pain, dispelling dampness, stopping diarrhea, and preventing itching; therefore, it can be applied to treat abdominal pain, toothache, vomiting, diarrhea, ascariasis, and other diseases caused by cold and dampness [15,16,17].

Nowadays, the pericarps of *Z. bungeanum* are clinically used as an important herbal medicine for the treatment of various painful diseases (including abdominal pain induced by cold and parasitic infestation, toothache, and bruises), vomiting diseases, edematous diseases, and itching diseases. Due to its extensive and defined pharmacological activities, over 30 kinds of prescriptions were listed in different versions of Ch.P, *Zhongyao chengfang zhiji*, and *Buban biaozhun*. The forms of these prescriptions include pill, tincture, oral liquid, powders, etc., among which pills are the most commonly used form (Table 1). In Western folk medicine, *Zanthoxylum* plants, as a traditional folk medicine, are commonly known as “toothache trees” due to their anesthetic or irritant properties, which renders them useful in the alleviation of acute and chronic pain [18].

Additionally, *Z. bungeanum* pericarps could be widely consumed as a popular spice and one of “eight essential condiments” in cooking because of their unique pungent flavor. Apart from the common application of pericarps, its leaves also have some medicinal value and health care function; the leaves are recorded in the books of Compendium of Materia Medica as pungent in flavor, warming in nature, with the capacity to clear food retention and remove moisture. The leaves can be used to treat indigestion, itching, and painful diseases. What is more, the pericarps and leaves of *Z. bungeanum* can be widely utilized in pickles, hotpots, and traditional Chinese cuisine in order to improve taste, and fresh young leaves can be used as a topping on dishes and sauces after mixing with soy bean paste. In addition, *Z. bungeanum* seed oil is often used as an antiphlogistic and diuretic, as well as for biodiesel production, and approximately 1 million metric tons are produced annually in China [22]. In addition, the root could be applied for the treatment of epigastric pains and bruises, eczema, and snake-bites [23].

## 3. Botany

**Figure 1:**
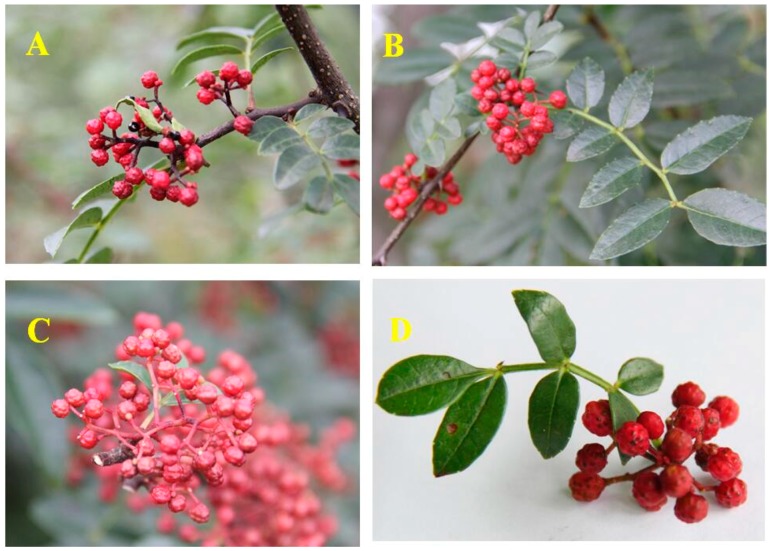
The stem (**A**); the leaves (**B**) and the pericarps (**C**,**D**) of *Z. bungeanum*.

*Z. bungeanum* (Figure 1) is a small deciduous shrub approximately 3–7 m in height. The stems of this plant are branched, 3–8 cm in diameter, woody and grey or brown and grey with hard prickles. The leaves are 1.5–7 cm in length, 1–3 cm in width, and are light green to dark green. The flowers are unisexual, white or pale yellow, and fragrant, and clustered in inflorescences; the inflorescences are acrogenous, branched, and approximately 2–6 cm in length; the rachises are covered with pubescence; the male flowers have 5–8 stamens and a 2-lobed rudimentary gynoecium; the female flowers have 2–3 carpels. The fruits are globular, follicle, 4–5 mm in diameter, scattered with numerous warty oil dots, red or purplish-red on the outer surface and yellowish on the inner surface; the fruit tastes pungent, has a numbing sensation in the mouth and the odour is fragrant; the fruits are harvested from August to October, the seeds and foreign matter are removed, and the fruits are then dried in the sun [17,24].

The distribution of *Z. bungeanum* is very wide due to its wide niche breadth. It is native to China and widely distributed in the provinces of Sichuan, Shaanxi, Yunnan, Guizhou, Gansu, etc., among which Sichuan province contains the largest production areas, famous for their high production and quality. In addition, it is also widely cultivated in the Japanese islands, the Korean peninsula, India, and other regions [25,26].

## 4. Phytochemistry

To the best of our knowledge, many chemical compounds have been isolated and identified from *Z. bungeanum* between the later 1880s and the present day. Currently, more than 140 constituents have been identified from this plant; furthermore, alkaloids and terpenoids have been identified as the characteristic components. This section details phytochemical studies that have been conducted on many parts of *Z. bungeanum*, including the stem, the leaf, the seed, the pericarps, and the roots. The identified compounds are listed in the following tables and the corresponding structures are also comprehensively presented.

### 4.1. Alkaloids *(**1**–**35**)*

#### 4.1.1. Alkylamides

**Figure 2:**
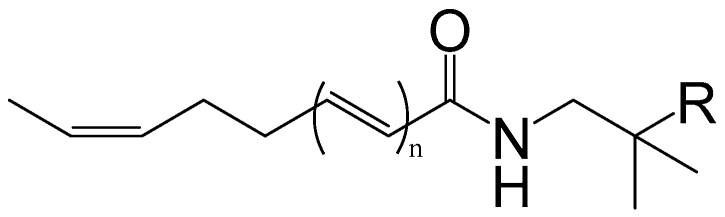
The minimum structure for the tingling sensation elicited by alkylamides.

**Figure 3:**
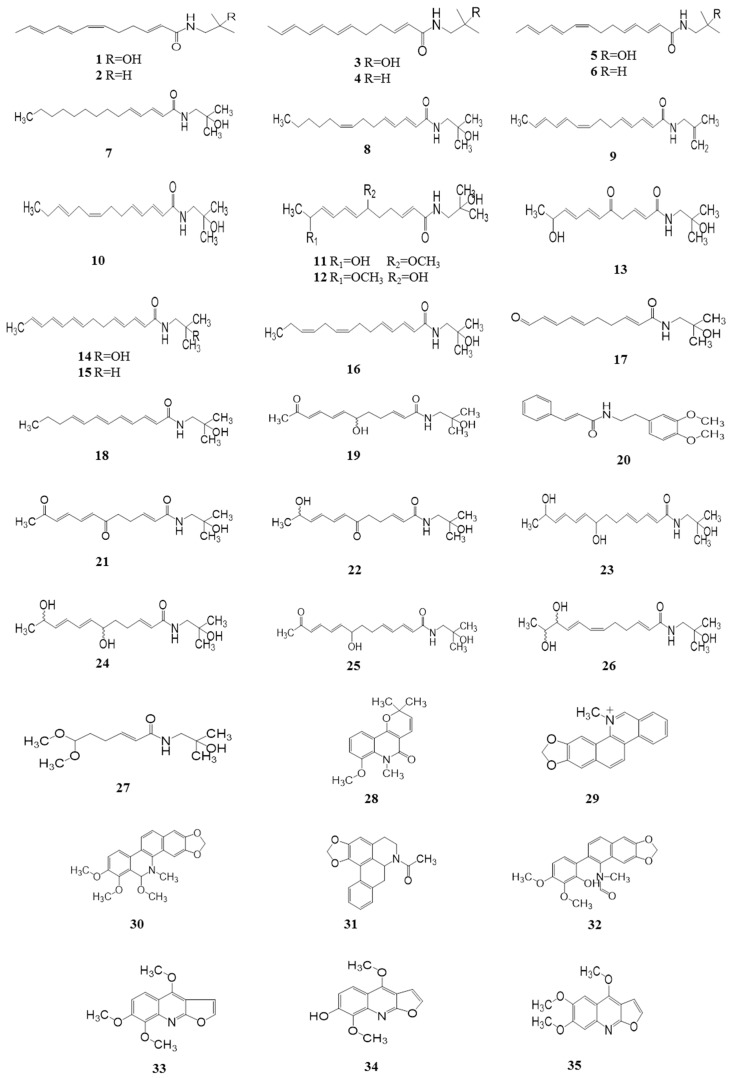
Chemical structures of alkaloids in *Z. bungeanum*.

Alkylamides, creating a strong numbing sensation in the mouth, are considered to be the main characteristic compounds. So far, more than 25 alkylamides have been isolated and identified from this plant. They are usually highly unsaturated with a unique taste because of the two or more conjugated double bonds. Hydroxy-α-sanshool (HAS), having four double bonds in the *cis*-configuration, is the active ingredient most responsible for the unique tingling sensation evoked by the pericarps of *Z. bungeanum*. HAS was first isolated from the pericarps of *Z. bungeanum* and identified by Yasuda et al. (1982) [27]. There are two different viewpoints on the unique sensation produced by HAS. In vitro, HAS has been shown to activate TRPV1 and TRPA1 in sensory neurons by influx of Ca^2+^ in cells [28]. Subsequently, Bautista et al. (2008) reported the activation of somatosensory neurons including small- and large- diameter cells elicited through the unique ability of HAS to inhibit two-pore potassium channels (KCNK3, KCNK9, and KCNK18) [18]. However, Hydroxy-β-sanshool (HBS) with four double bonds in the all *trans*-configuration has no effect when applied to the human tongue [29]. Additionally, Galopin et al. (2004) provided the minimum structure unit for the tingling sensation elicited by alkylamides (Figure 2). It is noteworthy that the all-*trans* alkylamides are tasteless, whereas the amides having a *cis* double bond are very pungent [30,31] (Table 2, Figure 3).

#### 4.1.2. Other Alkaloids

Apart from the alkylamides, there are also other alkaloids isolated from *Z. bungeanum*. To date, eight alkaloids isolated from *Z. bungeanum* have been reported. In 1981, zanthobungeanine, des-*N*-methylchelerythrine, 11-methoxychelerythrine, l-*N*-acetylanonanine, arnothianamide, and skimmianine were isolated from the roots of *Z. bungeanum* [32]. Moreover, haplopine and kokusaginine were also found to be present in the pericarps of *Z. bungeanum* in 1984 [33]. The chemical constituents of alkaloids and their corresponding structures are exhibited in Table 2 and Figure 3.

### 4.2. Terpenoids *(**36**–**103**)*

**Figure 4:**
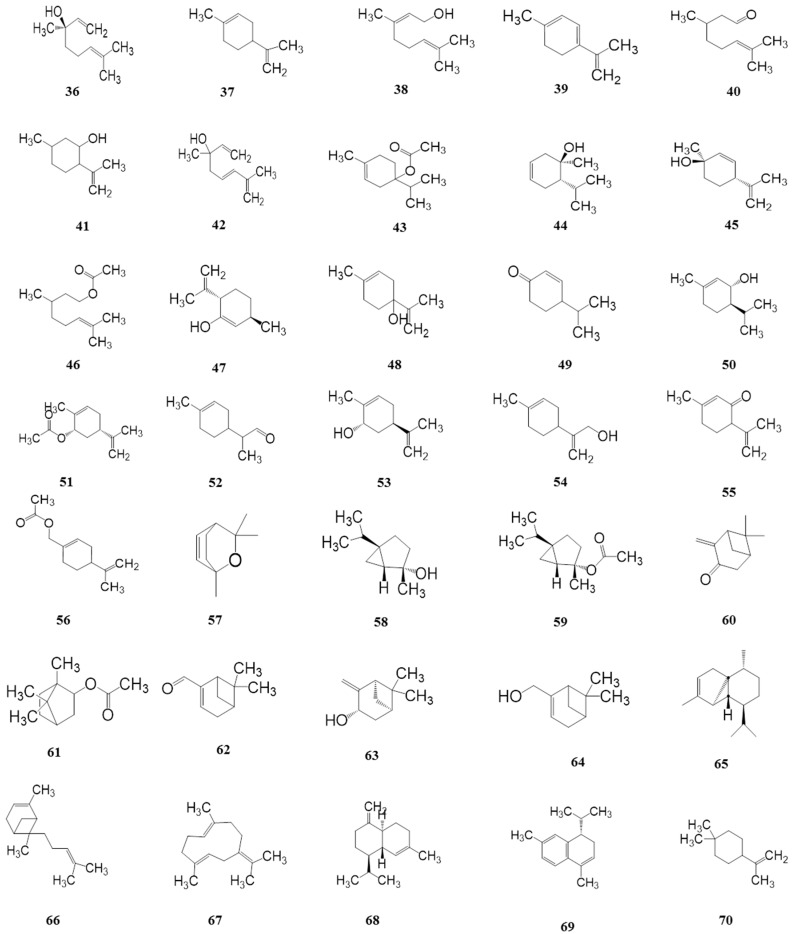

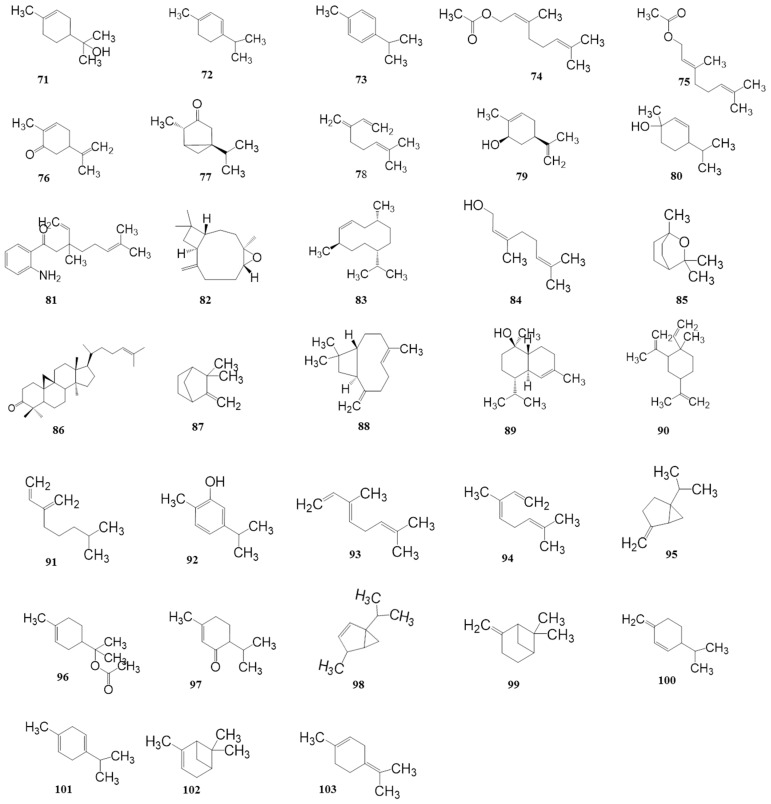
Chemical structures of terpenoids in *Z. bungeanum*.

Essential oils are the principle source of the special flavor in *HuaJiao*, and terpenoids are considered to be significant components due to their relatively high percentage among these compounds. To date, more than 65 constituents of terpenoids have been identified by gas chromatography coupled with mass spectrometry, which mainly consisted of high contents of monoterpenes and sesquiterpenoids. However, the chemical constituents and contents of terpenoids constituents are different in different studies, which can be explained by genetic characteristics, growth conditions, extraction methods, and other factors [39,40]. The terpenoids isolated from *Z. bungeanum* are presented in Table 3 and their corresponding structures are shown in Figure 4.

### 4.3. Flavonoids *(**104**–**129**)*

**Figure 5:**
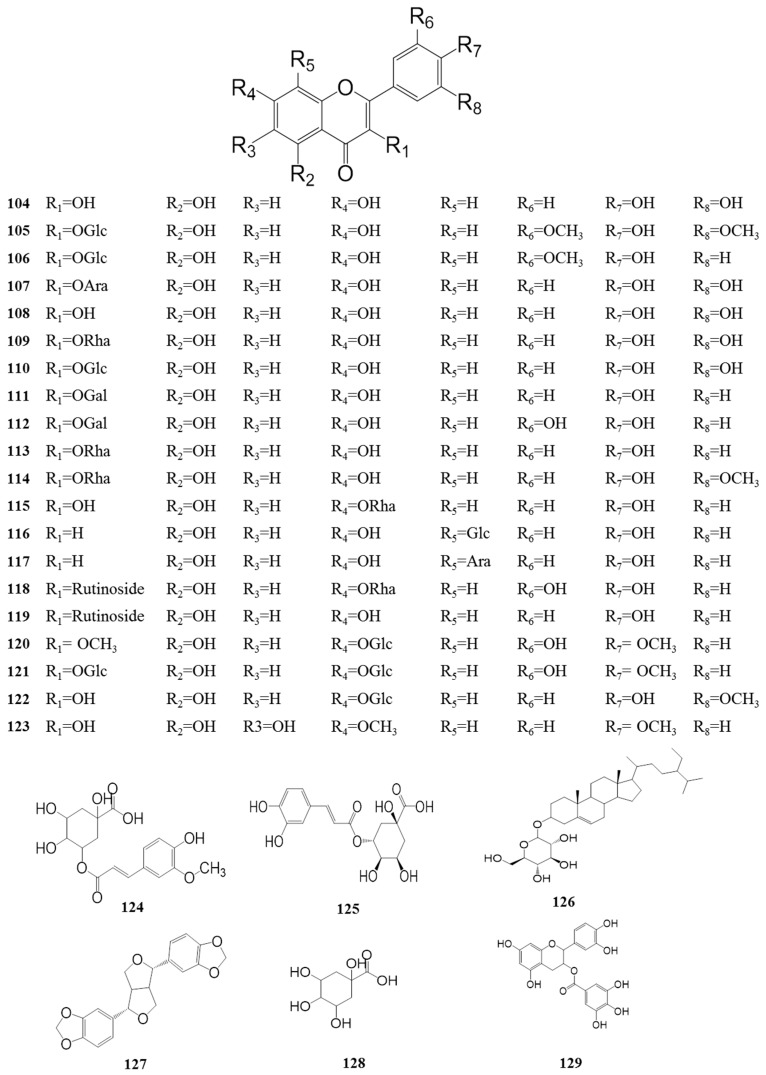
Chemical structures of the flavonoids in *Z. bungeanum*.

Flavonoids are common ingredients of numerous plants all over the world. Increasing the number of flavonoids isolated from *Z. bungeanum* has attracted much attention because of their broad range of pharmacological activities including antioxidant activity, antithrombotic activity, anti-aging activity, anti-tumor activity, etc. To date, more than 25 flavonoids, displayed in Table 4 and Figure 5, have been identified from this plant, such as quercetin, rutin, and quercetin 3-*O*-α-l-rhamnoside. Yang et al. (2013) and Zhang et al. (2014) provided significant data confirming that the leaves contain abundant flavonoids with prominent antioxidant abilities [10,23]. This could also explain the frequent addition of *Z. bungeanum* leaves to the Chinese diet for the promotion of human health.

### 4.4. Fatty Acids *(**130**–**139**)*

**Figure 6:**
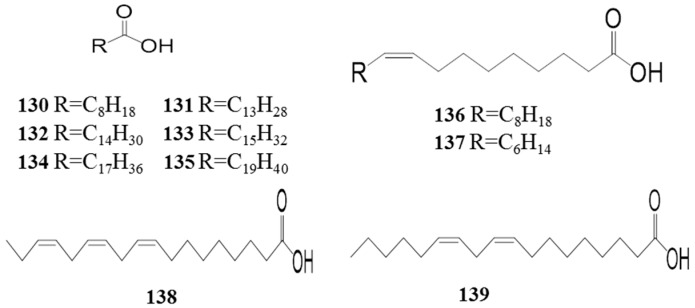
Chemical structures of the fatty acids in *Z. bungeanum*.

A few studies have been conducted investigating the fatty acids in *Z. bungeanum*. In 2007, palmitoleic acid was isolated from *Z. bungeanum* [47]; in addition, Xia (2011) reported that eicosoic acid, tetradecanoic acid, pentadecanoic acid, hexadecanic acid, oleic acid, and stearic acid were also present in the seeds of *Z. bungeanum* [11]. Lately, linolenic acid, linoleic acid, and nonanoic acid have been isolated and identified in *Z. bungeanum* [48,49]. As presented in Table 5 and Figure 6, the fatty acids isolated and identified from *Z. bungeanum* are mainly long carbon chains with a terminal carboxyl group.

### 4.5. Others *(**140**–**149**)*

**Figure 7:**
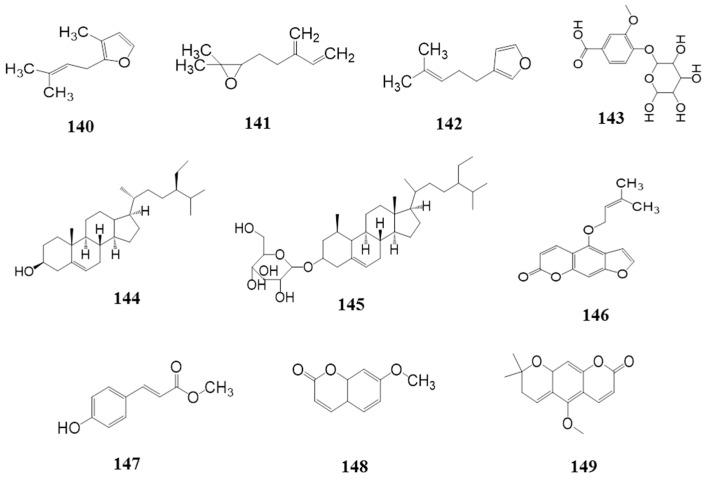
Other chemical structures in *Z. bungeanum*.

As shown in Table 6 and Figure 7, there are also some chemical compounds isolated from *Z. bungeanum* apart from the constituents listed above: rosefuran (**140**), mycrene epoxide (**141**), perillene (**142**), vanillic acid-4-glucoside (**143**), β-sitosterol (**144**), daucosterol (**145**), isoimperatorin (**146**), methyl-4-hydroxyphenylacrylate (**147**), 7-methoxycoumarin (**148**) and xanthoxylin (**149**).

## 5. Pharmacology

To the best of our knowledge, *Z. bungeanum* has been demonstrated to possess wide-reaching pharmacological effects, including effects on the digestive system, nervous system, and circulatory system, as well as anti-inflammatory and analgesic effects, antioxidant effects and anti-tumor effects, anti-fungal and antibacterial effects, insecticidal effects, and so on. In this section, the main pharmacology activities of *Z. bungeanum* are summarized and analyzed, as listed in Table 7.

### 5.1. Effect on the Digestive System

The characteristic pharmacological effect of *Z. bungeanum* on the digestive system has been comprehensively reviewed. The gastrointestinal smooth muscle in rabbits was stimulated by lower concentrations (4 mg/mL, intragastric (i.g.)) and depressed by higher concentrations (12 mg/mL, i.g.) of the water extracts of *Z. bungeanum* (WEZB), and this stimulating effect could be completely inhibited by atropine [51,52]. Furthermore, Zhang et al. (1991) reported that the WEZB (2.5, 5.0, and 10 g/kg, i.g., crude herb mass equivalent) showed significant inhibiting effects on experimental gastric ulcers in mice, including the pylorus ligation ulcer, water immersion stress ulcer, indomethacin-ethanol ulcer, and hydrochloric acid ulcer; additionally, they also found that the petroleum ether extracts of *Z. bungeanum* (PEEZB) could markedly inhibit diarrhea induced by castor oil (3.0 and 6.0 mL/kg, i.g.), while the WEZB (5 and 10 g/kg, i.g., crude herb mass equivalent) exhibited strong inhibition on diarrhea caused by senna leaf, which is different from the PEEZB [53]. Yuan et al. (2009) demonstrated that the essential oils of *Z. bungeanum* (EOZB) at doses of 0.1, 0.2, 0.4, and 0.8 mg/mL could dose-dependently inhibit the contraction of isolated duodenal smooth muscle of rabbits through a mechanism possibly associated with the blocking of the Ca^2+^ channel, calcium inward current, and release of intracellular calcium [54]. Meanwhile, a study also reported that the EOZB strongly inhibits the contraction of the colon smooth muscle in rabbits [55]. In addition, the WEZB (0.5, 1.0, and 2 g/kg, i.g., for 14 days) was reported to show great improvement in colonic shortening and body weight loss in dextran sodium sulfate (DSS)-induced experimental colitis in mice, whereas it decreased Disease activity index (DAI), a clinical parameter reflecting the severity of weight loss [56]. The mechanism may be related to the reduction of pro-inflammatory cytokines such as TNF-α, IL-β, and IL-12; additionally, suppression of NF-κB p65, IκBα phosphorylation, and the TLR4 pathway was also involved [56].

In addition to this, Kono et al. (2011) suggested that HAS, an active ingredient of *Z. bungeanum* pericarps, at doses of 0.3, 3.0, and 30 μmol/L, showed notable effects on improving the release of adrenomedullin (ADM) from intestinal epithelial cells in a dose-dependent manner [57]; in addition, they also showed that HAS (0.3 mg/kg) could markedly enhance the colonic blood flow in colitis rats. Additionally, HAS could also significantly evoke long-distance contraction (LDC) in vitro, and the mechanism may be associated with the blockage of the KCNK9 channel in the rat proximal colon (3, 10 and 30 μM) [58].

### 5.2. Effect on the Nervous System

It was reported that the EOZB and WEZB could reversibly inhibit the sciatic nerve impulse conduction of toads, and the required time of 20% EOZB and 20% WEZB for blocking the nerve impulse was approximately equal to that of procaine [59,60]. In addition, the polyphenol extracts from *Z. bungeanum* (PEZB) showed significant anti-depressive effects on behavioral despair models (induced by forced swimming and tail suspension) (50, 100, and 200 mg/kg, i.g.) and the underlying mechanism might involve the central monoaminergic systems [61]. Furthermore, previous studies have also demonstrated that the PEZB (50, 100, and 200 mg/kg, i.g., for 21 days) could improve climacteric depression caused by chronic unpredictable stress behavior. The mechanism for this effect might be related to the reduction of depressive symptoms, which are elicited by the regulation of the nerve-endocrine system [62]. In addition, it has been reported that the PEZB can upregulate the level of NE and 5-HT in the brain tissue of rats with post-stroke depression, and the mechanism may be associated with an inhibitory effect on monoamineoxidase (MAO) activity (50, 100, and 200 mg/kg, i.g., for 21 days) [63]. Additionally, the PEZB (50, 100, 200 mg/kg, i.g., for 14 days) also showed anti-depressive effects in an unpredictable stress model of depression in rats and an ovariectomized model of depression in mice [64,65].

One interesting study indicated that HAS (5 mg/kg, per os (p.o.)) can significantly shorten the escape latency in mice via a Morris water maze test, and this also exhibited a tendency of HAS to reduce the effect of scopolamine-induced dementia, which is probably mediated by the facilitation of ACh release [66]. Additionally, it is worth noting that gx-50, an active ingredient isolated from *Z. bungeanum*, could significantly enhance the cross-platform times (1.0 mg/kg, i.p., for 2 months), inhibit the release of cytokine induced by Aβ in microglia cells (2.5, 5, 10, and 20 μM), penetrate the blood–brain barrier, improve the cognitive abilities of mice in vivo (1.0 mg/kg, i.p., for 2 months), disassemble Aβ oligomers (5 μM), inhibit Aβ-induced neuronal apoptosis, increase bax expression, and reduce neuronal Ca^2+^ influx toxicity, which strongly suggests that gx-50 is a potential candidate drug for treating Alzheimer’s Disease (AD) [37,67]. In one report of 2016, eight isobutylhydroxyamides were isolated and identified from the pericarps of *Z. bungeanum*, and three of them—including qinbunamide, (10*RS*,11*SR*), and (10*RS*,11*RS*)-(2*E*,6*Z*,8*E*)-10,11-dihydroxy-*N*-(2-hydroxy-2-methylpropyl)-2,6,8-dodecatrienamide)—possess the ability to potentiate the activity of nerve growth factor (NGF) to stimulate neurite outgrowth from PC12 cells at concentration of 20 μM [26].

### 5.3. Effect on the Circulatory System

In 2005, the seed oil of *Z. bungeanum* (SOZB, 5, 10, and 20 mL/kg, i.g., for 4 weeks) was reported to have a notable effect on reducing cholesterol (CHOL) hyperlipidemia, triglyceride (TG), and low-density lipoprotein (LDL), as well as increasing high-density lipoprotein (HDL-C) [68]. Later, Liu et al. (2007) indicated that the SOZB (2.5 mL/kg, i.g., for 10 weeks) could decrease blood lipids and lower blood viscosity through reducing high blood viscosity (HBV), high low viscosity (HLV), CHOL, TG, and increasing HDL-C [69]. Furthermore, the SOZB at doses of 2.5, 5, and 10 g/kg also showed significant effect on reducing the serum levels of TG, total cholesterol (TC), low-density-lipoprotein cholesterol (LDL-C), malondialdehyde (MDA), and nitric oxide (NO) through the activation of PPARγ, which indicates that SOZB is a promising novel hypolipidemic health product [70]. In addition, previous studies have also demonstrated that the EOZB (2.0, 4.0, 6.0, 8.0, and 10.0 μL/mL) displays a dose-dependent relaxation of the contracted aortic muscle elicited by PE and KCl in rats, and the mechanism may decrease calcium influx and inhibit calcium channels [71].

Additionally, Yang et al. (2014) indicated that both alpha-linolenic acid (ALA) and its mixture with linoleic acid (ALA: linoleic acid = 1:1) isolated from *Z. bungeanum* seeds exhibited notable effects on the circulatory system. In vivo, after being treated with 250 mg/kg ALA, the survival rate of mice subjected to collagen-adrenaline-induced thrombosis was significantly increased by at least 3.5 times compared with the control mice. Furthermore, ALA and its mixture (70 and 175 mg/kg, p.o., for 10 days) could remarkably decrease platelet aggregation, and the mixture offered a better therapeutic effect than the pure ALA compound. In addition, the effects of ALA and its mixture (50, 100, and 250 mg/kg, respectively, p.o., for 10 days) on prolonged hemorrhage and coagulation time were also proven. The mechanism for anti-thrombosis effects may be related to the reduction in PI3K and Akt expression [72].

### 5.4. Anti-Inflammatory and Analgesic Effects

*Z. bungeanum* has a long history of usage in China as an anti-itching agent as well as for the treatment of pruritus vulvae-related diseases. In accordance with the traditional usage of *Z. bungeanum*, a few studies have demonstrated that these plants possess anti-inflammatory and analgesic effects both in vitro and in vivo. In 1994, the anti-inflammatory and analgesic abilities were evaluated through many animal models. The WEZB (2.5, 5.0, and 10 g/kg, i.g., for 3 days, crude herb mass equivalent) and the ether extracts (EEZB) (1.5, 3.0, and 6.0 mL/kg, i.g.) of *Z. bungeanum* exhibited significant anti-inflammatory and analgesic effects in animal models of inflammation (dimethylbenzene-induced ear oedema test and carrageenan-induced rat paw oedema in mice) and pain (acetic acid-induced torsion test in mice) [73]. Later, in 2010, the EOZB was also reported to be effective against dimethylbenzene-induced ear oedema and acetic acid-induced pain at doses of 0.05, 0.1, and 0.2 g/kg (i.g., for 14 days). At a dose of 0.1 g/kg, the WEZB showed notable activity with an inhibition ratio of 65.76% for oedema weight and 51% inhibitory value for the writhing responses [74].

Moreover, Shi et al. (2011) suggested that the alkaloids from *Z. bungeanum* (AZB) could inhibit hot-plate-induced pain (118, 236, and 472 mg/kg, i.g., for 3 days) and dextran-40-induced itch-scratch responses [75]. In addition, the methanol extracts of *Z. bungeanum* pericarps (MEZB) at a dose of 200 μM showed inhibitory effects in LPS-stimulated J774.1 macrophages and in LPS/IFN-γ-stimulated mouse peritoneal exudate macrophages via the inhibition of the expression of inducible nitric oxide aynthase (iNOS) mRNA. Furthermore, 4-*O*-β-d-glucopyranosyldihydroferulic acid isolated from the MEZB and identified by Thin-layer chromatography (TLC) behavior and the ^1^H-NMR spectrum, was reported to be effective in suppressing iNOS mRNA expression with an IC_50_ value of 6.5 μg/mL [76].

In another study of formalin-induced pain in rats, USV (ultrasonic vocalization) data was significantly lower than that of the control groups after the EEZB and the WEZB treatment, and the EEZB had better analgesic effect than WEZB. Additionally, the EEZB could relieve pain caused by warming similar to Lidocaine [77]. Additionally, the effects of HAS to quickly mediate pain were evaluated using recordings of cutaneous sensory fibres, whole-cell patch clamp, and calcium imaging. The results revealed that HAS (IC_50_ = 70 ± 7 μM) could inhibit the excitability of A*δ* mechanosensory nociceptors by blocking voltage-gated sodium channels to induce “fast pain” analgesia [78]. In addition, the seed oil of *Z. bungeanum* (SOZB) at doses of 0.5, 1.0, and 2.0 g/kg (i.g.) could markedly inhibit the dimethylbenzene-induced auricle edema with the inhibition rates of 68.52%, 70.43%, and 71.83% for the ear weight [48].

Apart from these, some compounds isolated from the pericarps of *Z. bungeanum* were found to exhibit notable anti-inflammatory effects by the suppression of nitric oxide (NO) production. Results revealed that the compounds ZP-amide D, ZP-amide E, ZP-amide F, and ZP-amide G showed inhibitory effects on nitric oxide (NO) production in LPS-stimulated RAW 264.7 macrophages, with IC_50_ values of 48.7 ± 0.32, 27.1 ± 1.15, 49.8 ± 0.38, and 39.4 ± 0.63 μM, respectively [38].

### 5.5. Antioxidant Effect

Lu et al. (1995) reported that the WEZB potentially has the ability to reduce the production of malondialdehyde (MDA) in hepatic tissue per gram [79]; in 2010, a study demonstrated that the flavonoids of water extraction, ethanol extraction, and acetone extraction from *Z. bungeanum* leaves (FWEZBL, FEEZBL, and FAEZBL) all had high scavenging activities against DPPH radicals with IC_50_ values of 24, 17.5, and 7.6 μg/mL, respectively [80]. Furthermore, the antioxidant effects on the SOZB obtained by the Box-Behnken design was measured by the scavenging activity towards the DPPH radical. The results showed that extraction pressure had a positive linear effect on the antioxidant activity, whereas extraction temperature had a negative linear effect [11]. A further investigation indicated that the ethanol extracts of *Z. bungeanum* leaves (EEZBL) and their sub-fractions (the ethyl acetate fraction, the acetone fraction, the acetone fraction, and the methanol fraction of the EEZBL) exhibited significant antioxidant effects on scavenging DPPH radical activity with low IC_50_ values of 40.75 ± 0.21, 13.20 ± 0.85, 18.55 ± 0.35, and 85.85 ± 2.19 μg/mL. In addition, the ethyl acetate fraction of the EEZBL was reported to possess the highest activity in the ferric reducing/antioxidant power assay (615.88 ± 1.86 μmol equiv. Trolox/g) and ABTS•^+^ radical cation decolorization assay (2147.83 ± 23.08 μmol equiv. Trolox/g) compared with the EEZBL and its sub-fraction [23,81]. Li et al. (2015) demonstrated that the polysaccharide of *Z. bungeanum* (PZB) showed prominent antioxidant effects towards Fe^3+^ (EC_50_ = 0.011 mg/mL), DPPH radical (EC_50_ = 0.021 mg/mL), chelating Fe^2+^ (EC_50_ = 0.056 mg/mL), and hydroxyl radical (EC_50_ = 0.008 mg/mL) in vitro [82]. At a dose of 10 mg/mL, the three fractions (<10, 10~30, and >30 kDa) of polypeptide from *Z. bungeanum* seeds kernel (PZBSK) displayed remarkable capacities for DPPH radicals with DPPH radical scavenging abilities of 53 ± 1.73, 40 ± 1.32, and 32 ± 0.70% [83].

In the treatment with the EEZBL (0.015%, 0.030%, and 0.045%, for 8 days), the hexanal content, the thiobarbituric acid reactive substances (TBARS) value, and the lipoxygenase (LOX) activity were remarkably lower during processing, both in the dorsal and ventral muscles [25]. In addition, salted fish with the EEZBL (0.018%) and the polyphenols (0.01% chlorogenic acid, hyperoside and quercitrin) had higher endogenous antioxidant enzyme (catalase, superoxide dismutase, and glutathione peroxidase) activities, lower peroxide value (PV), and thiobarbituric acid-reactive substance (TBARS) values than those of the control [84]. These findings showed that EEZBL can be regarded as a source of natural antioxidants.

Moreover, Zhang et al. (2014) revealed that some chemical compounds including quercetin, quercitrin, quercetin-3-*O*-β-d-glucoside, hyperoside, rutin, and isorhamnetin 3-*O*-α-l-rhamnoside possess strong scavenging activity against DPPH with IC_50_ values of 0.009 ± 0.001, 0.011 ± 0.001, 0.012 ± 0.001, 0.011 ± 0.001, 0.016 ± 0.001, and 0.028 ± 0.001 mM, respectively. Under peroxide stress, the protective effects of these compounds on *Escherichia coli* were also evaluated, and the results indicated that the cell growth rate increased to 1.88–5.76-fold in these compound-treated groups. Additionally, vitexin and quercitrin were found to have high inhibitory capacity on lipid peroxidation with lower IC_50_ values of 0.014 ± 0.001 and 0.013 ± 0.005 mM, respectively [23].

### 5.6. Anti-Tumor Effect

Recently, investigations into the anti-tumor effects of *Z. bungeanum* have been conducted. In vitro, the EOZB (4 mg/mL) showed significant anti-tumor effects on H22 with an inhibition rate of 76% at 72 h; in vivo, the EOZB (10, 25, 50, and 100 mg/kg) exhibited inhibitory effects on the growth of an established tumor in mice with inhibition rates of 20.15%, 40.03%, 60.25%, and 62.58%, respectively [85]. In addition, the EOZB displayed anti-tumor effects on HeLa (IC_50_ = 11.2 ± 0.2 mg/mL), A549 (IC_50_ = 6.26 ± 0.05 mg/mL), k562 (IC_50_ = 1.37 ± 0.03 mg/mL), and PC12 (0.5 mg/mL~2.0 mg/mL) in vitro [86,87]. Additionally, the EOZB (IC_50_ = 0.024%, for 48 h) has been reported to possess significant anti-proliferative effect towards HaCaT in a dose- and time-dependent manner. Furthermore, Western blot analysis suggested that the mechanism of these effects is involved with the increased expression of cleaved caspase-8/9/3, PARP, Bax, and decreased Bcl-2 levels. Additionally, the EOZB also could inhibit PC-3 cells, HEp-2 cells, Hela cells, and MFC-7 cells with IC_50_ values of 0.04%, 0.021%, 0.03%, and 0.023%, respectively [88]. In another investigation, sanshools could effectively suppress HepG2 cell proliferation (0~250 μg/mL) and induce apoptosis (0~200 μg/mL) in a time- and dose-dependent manner, and the mechanism mainly correlated with the upregulation of mRNA and protein expressions of p53 and caspase-3. In particular, the sanshools at a concentration of 250 μg/mL showed a notable inhibitory effect on HepG2 with inhibition rates of 51.2%, 73.01%, and 85.01% at 24, 48 and 72 h, respectively [89,90]. Zhao et al. (2017) revealed that the EEZB (1 μg/mL~8 μg/mL, for 48 h) could dose-dependently induce obvious apoptosis and inhibit cell growth in HepG2 cells, and the main possible mechanism is to inhibit cell proliferation and induce cell apoptosis through downregulating Mcl-1, Survivin, Bcl-xL expression and upregulating Bax expression [91].

Besides, some bio-active ingredients isolated from *Z. bungeanum* also exhibited anti-tumor effects. Hyperoside isolated from *Z. bungeanum* leaves displayed inhibitory effects against SW620 cells with the half maximal inhibitory concentration values of 72.35 ± 5.46, 36.41 ± 1.36, and 19.51 ± 4.95 μM for 24, 48, and 96 h, respectively. Further investigations revealed that the mechanism of these effects may be associated with the increased production of reactive oxygen species (ROS), reduced (mitochondrial membrane potential) ΔΨm, upregulation of X protein, cytochrome c, caspase 9, apoptotic protease activating factor 1, and caspase 3, and the inhibition of the mRNA expression levels of glutathione peroxidase (GSH Px) and catalase (CAT) [92]. Apart from these, the major chemical compounds of the EOZB including d-limonene (IC_50_ = 0.009%, *v*/*v*, for 48 h), terpinen-4-ol (IC_50_ = 0.028%, *v*/*v*, for 48 h), and β-myrcene (IC_50_ = 0.013%, *v*/*v*, for 48 h) have been proven to significantly inhibit the proliferation of HaCaT cells [88].

### 5.7. Anti-Bacterial and Anti-Fungal Effects

In 1995, after treatment with the WEZB (5 mg/mL, for 4 days, crude herb mass equivalent), the bacteria count of *Staphylococcus aureus* and *E. coli* was significantly decreased compared with the control group, and the anti-bacterial activity of the WEZB on *S. aureus* was stronger than *E. coli* [79]. Later, the study of Gong et al. (2009) reported the chemical components of the EOZB, and the inhibitory activity against plant pathogenic fungi was evaluated by the mycelial growth inhibition method and the values of IC_50_. The results indicated that the EOZB had a broad spectrum of activity against phytopathogenic fungi including *Alternaria solani*, *Botryodiplodia theobromae*, *Fusarium oxysporum* f.sp. *cucumerinum*, *Fusarium oxysporum* f.sp. *niverum*, *Bipolaris maydis*, *Leptosphaeria maculans*, *Magnaporthe grisea*, *Rhizoctonia cerealis*, *Rhizoctonia solani*, *Venturia pirina*, and *Verticillium dahliae* with low IC_50_ values of 0.44, 0.48, 0.43, 0.48, 0.24, 0.13, 0.28, 0.27, 0.24, 0.41, and 0.32 mg/mL, respectively. Furthermore, the EOZB was also found to strongly inhibit the growth of *R. solani* and *R. cerealis* mycelia with IC_50_ values of 0.95 and 1.22 mg/mL, respectively [43]. In addition, the EOZB also showed significant inhibitory effects towards *Bacillus subtilis*, *Salmonella*, *Staphylococcus aureus*, *Bacillus cereus*, *E. coli*, *Proteus vulgaris*, *Pentcillum Crtinum Thom*, *Aspergillus flavus*, *Aspergillus niger*, *Ruinous orgzae*, and *Saccharomyces cerevisiae* with minimum bacteriacidal (or fungicidal) concentrations of 25.0, 6.25, 25.0 , 12.5, 12.5, 12.5, 12.5, 12.5, 12.5, 25.0, and 12.5 mL/L, respectively [93]. Zhu et al. (2011) suggested that the EOZB could be used as an antibacterial agent in the food industry and for the inhibition of food-borne bacteria [44]. Five common food-borne bacteria—*S. aureus*, *Bacillus subtilis*, *Bacillus cereus*, *Bacillus laterosporus laubach*, and *E. coli*—were inhibited by the EOZB with minimum inhibitory concentrations (MIC) of 5.0, 1.25, 2.5, 1.25, and 2.5 mg/mL, while the minimum bactericidal concentrations (MBC) were 20, 2.5, 10, 5.0, and 5.0 mg/mL, respectively. In vitro, the EOZB (MIC = 6.25%) and α-pinene (MIC = 12.50%) showed strong inhibition against *Fusarium sulphureum* with decreased spore germination and germ tube elongation, and declined cell membrane integrity. Furthermore, in vivo testing showed that the EOZB (6.25%) and α-pinene (12.50%) effectively reduced the lesion diameter of potato inoculated with *F. sulphureum* [40].

Apart from these, the antibacterial activity of the ethanol extracts and their five fractions from *Z. bungeanum* leaves against five tested plant pathogenic fungi were assayed. IC_50_ values of the EEZBL against *Botrytis cinerea*, *Piricularia oryzae*, *Physalospora piricola*, *Glomerella cingulate*, and *Venturia pyrina* were determined to be 11.82 ± 1.15, 12.31 ± 0.45, 39.48 ± 2.25, 13.00 ± 1.34, and 33.22 ± 3.61 mg/mL, while the chloroform fraction of the EEZBL against five tested pathogenic fungi with EC_50_ values were determined to be 9.39 ± 0.07, 4.18 ± 0.08, 10.89 ± 1.62, 0.83 ± 0.24, and 5.35 ± 0.34 mg/mL, respectively [81]. In addition, the ethyl acetate fraction and acetone fraction of the EEZBL also showed notable inhibitory activity against *B. cinerea*, *P. oryzae*, *P. piricola*, *G. cingulate*, and *V. pyrina* [81].

### 5.8. Insecticidal Effects

Bowers et al. (1993) reported that three active monoterpenes isolated from *Z. bungeanum*—piperitone, 4-terpineol, and linalool—exhibited high repellent activity against ants, with 80% of the ants repelled from feeding on the sucrose solution of 8.91, 14.13, and 20 μg/cm^2^ [94]. It was reported that the EOZB obtained by supercritical CO_2_ had high insecticidal activity against *Sitophilus zeamais* and *Tribolium castaneum* [95]. In addition, three fractions of the EOZB (petroleum ether fraction, dichloromethane fraction, and diethyl ether fraction) showed significant insecticidal effects towards *T. castaneum* with LD_50_ values of 0.0713, 0.11699, and 0.12267 μL [95]. In addition, Kou (2015) suggested that the EOZB obtained by steam distillation displayed strong anti-insect activity against *aedes albopictus* at doses of 15, 25, 35, and 45 μg/mL [96]. Furthermore, *T. castaneum* could be killed by the MEZB at doses of 0.5, 1.0, and 1.5 mg/mL [96]. Additionally, two EOZB samples were obtained from the pericarps of *Z. bungeanum* with the methods of hydrodistillation (HD) and supercritical fluid CO_2_ extraction (SFE), and their bioactivities against *Lasioderma serricorne* adults were evaluated. The results indicated that the SFE sample and HD sample showed significant anti-insect activity against *Lasioderma serricorne* adults with LC_50_ values of 3.99 μg/mL and 12.54 μg/mL [41].

Apart from these, it was revealed that some major chemical components of the EOZB (eucalyptol, limonene, γ-terpinene, linalool, *α*-terpineol and 4-terpinenol) played a key role in anti-insect activity against *L. serricorne* with LC_50_ values of 5.18, 14.07, 12.01, 18.04 3.27, and 6.90 mg/L. Moreover, these chemical compounds also exhibited strong contact toxicity against *L. serricorne* with LD_50_ values of 15.58, 13.66, 14.19, 12.74, 11.99, and 8.62 μg/adult, respectively [41].

### 5.9. Other Pharmacological Effects

In addition to the pharmacological effects listed above, *Z. bungeanum* also exhibited other effects. The sanshools isolated from the pericarps of *Z. bungeanum* were regarded as a topical lifting agent for wrinkles based on their capacity to relax subcutaneous muscles [97]. Furthermore, the sanshools (0.8 mg/mL) of *Z. bungeanum* elicited protective effects on rice-seedling growth, chlorophyll content, and root activity in rice seedlings exposed to metolachlor; this activity was related to the upregulated expression of four Glutathione transferases (GST) genes, especially representative GST genes (OsGSTU3) [98]. Tang et al. (2014) evaluated the anti-asthma effects of the seeds of *Z. bungeanum* (SZB), and they found that the SZB at doses of 0.25, 0.5, and 1.0 g/kg could obviously prolong the latency period of induced asthma (LPIA) and reduce the frequency of citric acid-induced cough compared with that of vehicle treatment. Moreover, the SZB (0.5, 1.0, and 2.0 g/kg, i.g.) also showed anti-fatigue and anti-anoxia ability [48]. Additionally, Lan et al. (2014) demonstrated that the EOZB (3%) could significantly enhance the percutaneous absorption of drugs with different lipophilicities [46]. Meanwhile, limonene (3%) exhibited the highest permeation fluxes and cumulative amounts in comparison with terpinen-4-ol (3%) and 1,8-cineole (3%). The mechanism of these enhancers which promoted the skin permeation of drugs may be associated with the effect on skin stratum corneum (SC) lipids [45].

Apart from these, the *n*-butanol fraction isolated from the EEZB has effects on the cholesterol metabolism. In an in vivo study, it (50 and 200 mg/kg/day, i.g., for 4 weeks) can inhibit hyperlipidemia effects with decreased serum TC and TG levels in apoE-ko mice. In an in vitro study, with the *n*-butanol fraction of the EEZB treatment (0.05, 0.1, and 0.2 mg/mL), the TC, TG, FC (free cholesterol) levels and apolipoprotein B (apoB) secretion were significantly decreased in HepG2 cells exposed to sterols and 25-hydroxycholesterol, whereas apolipoprotein A1 (apoA1) secretion was increased [99]. The mechanism may be related to the increase in low density lipoprotein receptor (LDLR) protein and inhibition of the expression of hydroxy methylglutaryl coenzyme A reductase, HMGCR [99].

### 5.10. Summary of Pharmacologic Effects

In conclusion, *Z. bungeanum* has an extensive range of pharmacological effects which includes effects on the digestive system, effects on the nervous system, effects on the circulatory system, anti-inflammatory and analgesic effects, anti-bacterial and anti-fungal effects, as well as antioxidant effect and anti-tumor effects, etc. These pharmacological activities mainly have focused on the extraction or preparations of *Z. bungeanum*, which indicates that this plant has a promising potential for treating disease. However, there are few systemic investigations regarding the individual compounds and their corresponding pharmacological activities, as well as action mechanism. Therefore, future research into pharmacological effects, structure-function relationships, and mechanisms of the plant’s bio-active components should be explored by in vivo and in vitro experiments.

## 6. Pharmacokinetics

To date, there are few pharmacokinetics studies of the extracts and compounds of this plant. Previous pharmacokinetics studies of *Z. bungeanum* mainly focused on its alkylamides including hydroxyl-α-sanshool (HAS), hydroxyl-β-sanshool (HBS), hydroxyl-γ-sanshool (HRS) and other sanshools.

After oral administration of the WEZB at a dose of 1.3 g/mL, the peak time (Tmax) and peak plasma concentration (Cmax) values were determined to be 30.0 min and 46.720 g/kg, respectively, and the t1/2 of the WEZB was 79.26 min. Furthermore, the area under the concentration-time curve (AUC) was also determined, and the AUC_0__–t_ was 102.015 g/h/kg [100]. Furthermore, Fang et al. (2014) reported that the t1/2 values of alkylamides were determined to be 179.33, 118.03, 134.01, and 241.51 min of different intestinal segments of rats (including duodenum, jejunum, ileum, and colon), and the jejunum was regarded as the best absorption site of alkylamides [101].

In addition, a simple, rapid, and sensitive UHPLC–MS/MS method was developed for the determination of HAS, HBS, and HRS concentration in rat plasma. After the subcutaneous administration of the EEZB at a dose of 11.0 mg/kg (equivalent to 6.21 mg/kg of HAS, 1.36 mg/kg of HBS and 0.32 mg/kg of HRS), the peak times (Tmax) of HAS, HBS, and HRS were determined to be 36, 42, and 69 min, respectively, and the peak plasma concentration (Cmax) values were 1468, 432, and 41.49 ng/mL, respectively. Moreover, the area under the concentration-time curve (AUC) was also determined, and the AUC_0__–t_ of HAS, HBS, and HRS were 3816, 819, and 147 ng/mL, respectively; additionally, the AUC_0__–∞_ were 3890, 839, and 160 ng/mL, respectively. Meanwhile, pharmacokinetics studies of EOZB (4.4 mg/kg) after intravenous injection were also conducted. The Cmax values of HAS, HBS, and HRS were 1215, 324, and 34.70 ng/mL, respectively, and the t1/2 were 65.4, 91.2, and 99.6 min, respectively, and the AUC_0__-t_ were 1498, 385, and 65 ng/mL, respectively, and the AUC_0-∞_ were 1551, 441, and 72 ng/mL, respectively. In addition, the subcutaneous absolute bioavailability were 100.2, 76.2, and 90.3% for HAS, HBS, and HRS, respectively [102].

Apart from these, the study of gx-50 (20 mg/kg, p.o.) on metabolism was also determined by LC-MS/MS. The results demonstrated that gx-50 could be absorbed into the blood and penetrate the blood–brain barrier (BBB) after oral administration, and it immediately distributed to brain tissue (5 min post-per os (PO)) and was finally excreted approximately 4 h post-PO [37].

## 7. Toxicology

For thousands of years, *Z. bungeanum* was commonly considered to be a traditional Chinese medicine with low toxicity [15]. To date, investigations regarding the toxicities of *Z. bungeanum* are scarce, and previous studies mainly focus on its extracts (Table 8). In 1995, Tong et al. (1995) demonstrated that the median lethal concentration (LD_50_) value of the WEZB in mice was 45 g/kg (i.g., crude herbs mass equal) [16]. However, a report in 2010 suggested that the LD_50_ value of the WEZB in mice was 51.14 g/kg (crude herbs mass equal, i.g.), and this can be explained by the toxicity of *Z. bungeanum* which varied with the genetic characteristics, growing conditions, and medicinal materials [100]. Furthermore, Zhao et al. (2003) studied the toxicity of the WEZB on the viscera of mice, and the WEZB (0.5, 1.0, 2.0, and 4 g/kg, i.g., crude herbs mass equal) showed low toxicity on liver including ballooning degeneration, cytoplasm rarefaction, and some spotty necrosis [103]. In a cell-based model, the WEZB was added to J774.1 cells, and the results showed that the WEZB had no toxicity at doses of 100, 200, and 400 μg/mL [56].

The toxicity of the EOZB has also been investigated in recent years. The LD_50_ value of different approaches including intragastric administration (i.g.), intraperitoneal injection (i.p.), intramuscular injection (i.m.), and hypodermic injection (i.h.) of the EOZB was determined to be 2.27, 2.03, 4.64, and 5.32 g/kg, respectively [104]. After treatment with the lethal dose of EOZB, drowsiness, diarrhea, arrhythmia, twitchy limbs, and even death were observed in mice. Moreover, it has been reported that the EOZB resulted in low toxicity in both HaCaT cells and CCC-ESF-1 cells with a dose-dependent decrease in cell viability at IC_50_ values (i.e.) of 2.435 and 3.649 mg/mL, respectively [45].

In conclusion, *Z. bungeanum* showed low toxicity potential for use as a flavoring and traditional Chinese medicine. The occurrence of adverse reaction to *Z. bungeanum* mainly resulted from the over-dosage or misuse of *Z. bungeanum*. Obviously, the over-dosage of these plants will cause severe adverse reactions or even death. Furthermore, certain people should take *Z. bungeanum* with caution, such as pregnant women and those with Yin deficiency [15].

## 8. Future Perspectives and Conclusions

In summary, *Z. bungeanum* Maxim. has been used in Asian countries for many years, and many kinds of chemical constituents have been isolated and identified from this plant. There is no doubt that *Z. bungeanum* is an important and effective traditional Chinese medicine and food additive with a long history. Significant breakthrough has been made on multiple aspects of this plant in the past decade. However, it is worthy to note that there are still several challenges tha require further investigation to satisfy clinical research.

First, pharmacological studies have mainly focused on crude extracts and preparation, and there is not sufficient evidence to explain the special action mechanism for the pharmacological activity of this plant. Therefore, further investigation should be carried out to study the bioactive compounds and its action mechanism and structure-function relationship. Second, there are not enough studies regarding the pharmacokinetics and clinical research of *Z. bungeanum*, and few evaluations of the toxicity on a cellular and molecular level have been explored. Thus, future study of *Z. bungeanum* should focus more on the pharmacokinetics study of other constituents besides alkylamides, and toxicity studies should be performed on its molecular and cellular level to investigate any side effects in clinical research. Third, according to the current in vivo and in vitro investigation, HAS is the main active compound responsible for the special taste and diverse pharmacological activity. However, HAS is not stable in normal storage conditions and it may be sensitive to oxygen due to the conjugated triene system [9]. Therefore, more stable derivatives of HAS and other sanshools should be synthesized by modification of the structure. Fourth, traditional usages of *Z. bungeanum* include few prescriptions, and the processed products of *Z. bungeanum* are becoming fewer and fewer. Therefore, new prescriptions and products need to be developed to meet the clinical requirement. Fifth, the study of leaves, seeds, stems, and roots in *Z. bungeanum* has been relatively slow in comparison with research of pericarps, and it is necessary to investigate the chemical compounds and pharmacological activity of every part of *Z. bungeanum* to ensure the full utilization of the possible medicinal usages of this plant. Sixth, because of its rich biodiversity and morphological similarity to the *Zanthoxylum* genus, it is difficult to identify *Z. bungeanum* and its related plants. Thus, it is important to find a potential strategy that can control the quality of this plant and establish a unified international quality evaluation system. Lastly, due to its wide distribution and cultivation in many areas of the world, it is very important to enhance the efficiency and quality of the picking process.

The current literature provides a full-scale review on the progress of traditional uses, botany, phytochemistry, pharmacology, pharmacokinetics, and toxicology of *Z. bungeanum*, and proposes some issues worth investigating in the future, which will greatly facilitate the comprehensive understanding and effective development of *Z. bungeanum* treaments and applications.

## Figures and Tables

**Table 1:** The traditional and clinical use of *Z. bungeanum* in China.

Preparation Name	Main Compositions	Traditional and Clinical Usages	Ref.
Hua Zheng Hui Sheng Tables	Pericarpium *Zanthoxyli*, Herba *Leonuri*, Flos *Carthami*, Radix *Angelicae Sinensis*, Lignum *Sappan*, Rhizoma *Chuanxion*, *Ginseng radix* and *rhizome*, *Lignum Dalbergiae Odoriferae*	Removing blood stasis, curing blood accumulation, postpartum blood stasis	[7]
Wu Mei Pills	Pericarpium *Zanthoxyli*, *Ginseng radix* and *rhizome*, Radix *Angelicae Sinensis*, Rhizoma *Zingiberis*, Fructus *Mume*, Herba *Asari*, Rhizoma *Coptidis*, Cortex *Phellodendri*, Radix *Aconiti Lateralis* Preparata	Clearing the liver, regulating the middle burner, clearing the upper burner, warming the lower burner, and curing ascariasis, chronic dysentery, Jue Yin headache	[7]
Quan Lu Pills	Pericarpium *Zanthoxyli*, *Cornu Cervi Pantotrichum*, Herba *Cynomorii*, Radix *Bupleuri*, Radix *Rehmanniae*, Radix *Achyranthis Bidentatae*, Radix *Rehmaniae* Praeparata, *Myrrha*, *Semen Cuscutae*, Fructus *Lycii*	Invigorating the kidney and essence replenishment, invigorating the spleen and supplementing Qi, and curing weak waist, cold chills, deafness, tinnitus	[7]
Bo Yun Tui Yi Pills	Pericarpium *Zanthoxyli*, Flos *Buddlejae*, Fructus *Tribuli*, Flos *Chrysanthemi*, Herba *Equiseti Hiemalis*, *Feriostracum Serpentis*, Herba *Schizonepetae*, Fructus *Viticis*, Herba *Menthae*, Radix *Angelicae Sinensis*, Rhizoma *Chuanxion*	Cleaning heat, removing wind, and improving eyesight and curing blurred vision caused by wind-heat	[7]
Tong Luo Qu Tong Gao	Pericarpium *Zanthoxyli*, Radix *Angelicae Sinensis*, Rhizoma *Chuanxion*, Flos *Carthami*, Fructus *Piperis*, Flos *Caryophylli*, Cortex *Cinnamomi*, Fructus *Piperis Longi*, Rhizoma *Zingiberis*, *Camphora*, *Borneolum Syntheticum*	Promoting blood circulation, removing meridian obstruction, dispelling cold, removing dampness, relieving swell and pain, and curing blood stagnation as well as cold dampness blocking in collaterals	[7]
Kang Fu Ruan Gao	Pericarpium *Zanthoxyli*, Radix *Angelicae Dahuricae*, Fructus *Cnidii*, Radix *Inulae*, *Borneolum Syntheticum*	Curing pruritus valvae, leucorrhea disease, itching	[7]
Zi Hua Shao Shang Ruan Gao	Pericarpium *Zanthoxyli*, Radix *Arnebiae Seu Lithospermi*, Radix *Rehmanniae*, Prepared Radix *Rehmanniae*, *Borneolum Syntheticum*, Rhizoma *Coptidis*, Radix *Glycyrrhizae*, Rhizoma *Coptidis*, Radix *Angelicae Sinensis*	Curing burning and scalding disease	[7]
Chang Chun Yao Jiu	Pericarpium *Zanthoxyli*, Rhizoma *Atractylodis*, Prepared Radix *Rehmanniae*, Fructus *Gardeniae*, Fructus *Amomi Rotundus*, Herba *Epimedii*, Radix *Achyranthis Bidentatae*, *Psoralea corylifolia Linn*, Radix *Paeoniae Alba*, Cortex *Eucommiae*	Curing backache caused by deficiency of the kidney, rheumatism, debility, and weak blood	[19]
Qian Zi Hong Ke Li	Pericarpium *Zanthoxyli*, *Geranium strictipes Knuth*, Herba *Senecionis Scandentis*, Cortex *Schizophragmatis Integrifolii Radicis*, Radix *Myricae Rubrae*	Curing dysentery, diarrhea caused by summer heat, dampness, and dyspepsia	[19]
Shen Rong Gu Ben Huan Shao Pills	*Pericarpium Zanthoxyli*, *Ginseng radix andrhizome*, *Radix Aconiti Lateralis Preparata Cornu Cervi Pantotrichum*, *Cortex Cinnamomi*, *Semen Cuscutae*, *Cortex Eucommiae*, *Herba Epimedii*, *Radix Achyranthis Bidentatae*, *Actinolitum*	Reinforcing the kidneys to strengthen Yang, strengthening tendons and bones, benefiting Qi	[19]
Zhuang Yuan Bu Xue Pills	Pericarpium *Zanthoxyli*, Radix *Angelicae Sinensis*, *Colla Corii Asini*, Radix *Morindae Officinalis*, *leopard bone*, Fructus *Psoraleae*, Semen *Plantaginis*, *Halloysitum Rubrum*, Radix *Achyranthis Bidentatae*, Cortex *Lycii*, *Radix Rehmanniae*, Cortex *Eucommiae*, *Poria*	Building fitness, calming nerves, reinforcing the stomach, and curing weak waist, fatigue, insomnia, forgetfulness, poor appetite, and loose stools	[20]
Hui Chun Jiu	Pericarpium *Zanthoxyli*, Radix *Angelicae Sinensis*, Rhizoma *Atractylodis*, Cortex *Lycii*, Flos *Caryophylli*, Cortex *Eucommiae*, *Poria*, Radix *Aconiti Lateralis* Preparata, Radix *Glycyrrhizae*, Radix *Aucklandiae*	Nourishing Yin, tonifying Yang, reinforcing the vital essence, benefiting Qi, nourishing the blood and curing spiritual burnout, weak waist, loss of appetite	[20]
Zhi Chuang Wai Xi Yao	Pericarpium *Zanthoxyli*, Rhizoma *Coptidis*, Radix *Saposhnikoviae*, Radix *Glycyrrhizae*, *Natrii Sulfas*, *Galla Chinensis*, Herba *Houttuyniae*	Removing poison, relieving itching, swelling and pain, and curing hemorrhoids, anal pain, swelling and stiffness	[20]
Ke Tong Ding	Pericarpium *Zanthoxyli*, *Oleum Menthae*, Radix and *Rhizoma Litseae Cubebae*, Rhizoma *Curcumae*, Rhizoma *Alpiniae Officinarum*, Herba *Pogostemonis*, Radix *Scutellariae*, Lignum *Dalbergiae Odoriferae*	Dispelling wind and dampness, promoting blood circulation, alleviating pain, and curing punch injury, rheumatism	[20]
Wen Shen Quan Lu Pills	Pericarpium *Zanthoxyli*, Radix *Angelicae Sinensis*, Rhizoma *Chuanxion*, Flos *Carthami*, Radix *Morindae Officinalis*, Rhizoma *Atractylodis Macrocephalae*, Fructus *Psoraleae*, Lignum *Aquilariae Resinatum*, Pericarpium *Citri Reticulatae*, Radix *Achyranthis Bidentatae*, Radix *Codonopsis*, Radix *Rehmanniae*, Radix *Glycyrrhizae*	Warming the kidneys, reinforcing Qi, nourishing blood, and curing dizziness, forgetful, tinnitus, weak waist, ennui, impotence	[20]
Jian Shen Quan Lu pills	Pericarpium *Zanthoxyli*, Radix *Angelicae Sinensis*, Rhizoma *Atractylodis Macrocephalae*, Fructus *Psoraleae*, Lignum *Aquilariae Resinatum*, Pericarpium *Citri Reticulatae*, Rhizoma *Chuanxiong*, Radix *Rehmanniae*, *Poria*, Radix *Glycyrrhizae*, Radix *Astragali*	Nourishing blood, reinforcing Qi, warming the kidneys, curing weak waist, mental exhaustion caused by blood deficiency	[20]
Chan Ma Zhen Tong Ding	Pericarpium *Zanthoxyli*, Herba *Asari*, *notoginseng* radix and rhizome, *Venenum Bufonis*, Semen *Serychni*, Fructus *Evodiae*, Radix *Aconiti Kusnezoffii*, Folium *Sinapis*, Fructus *Gleditsiae*, *Camphora*	Relaxing tendons, activating collaterals, promoting blood circulation, removing blood stasis, and curing paining joints, muscle injury, periathritis of the shoulder, hyperosteogeny	[21]
Suan Tong Pen Wu Ji	Pericarpium *Zanthoxyli*, Lignum *Sappan*, Radix *Aconiti Kusnezoffii*, Radix *Aconiti*, Radix *Angelicae Pubescentis*, Rhizoma and Radix *Notopterygii*, Fructus *Liquidambaris*, Fructus *Chaenomelis*, Rhizoma *Arisaematis*, Rhizoma *Pinelliae*	Relaxing tendons, activating collaterals, dispelling wind, alleviating pain, and curing sprain, repetitive strain injury, aching muscles	[21]
Zhen Tong Huo Luo Ding	Pericarpium *Zanthoxyli*, Radix *Aconiti Kusnezoffii*, Rhizoma *Pinelliae Radix Aconiti*, *Camphora*, Fructus *Gardeniae*, Radix and Rhizoma *Rhei*, Fructus *Chaenomelis*, Rhizoma *Arisaematis*, Rhizoma and Radix *Notopterygii*, Radix *Angelicae Pubescentis*	Relaxing tendons, activating collaterals, dispelling wind, alleviating pain, and curing eriathritis of the shoulder, hyperosteogeny, arthritis, cervical spondylopathy	[21]
An Wei Zhi Tong San	Pericarpium *Zanthoxyli*, *Os Sepiae*, Fructus *Foeniculi*, Concha *Margaritifera Usta*, Cortex *Cinnamomi*, Rhizoma *Zingiberis*, Rhizoma *Kaempferiae*, Radix and Rhizoma *Rhei*, Flos *Caryophylli*, Pericarpium *Citri Reticulatae*, *Oleum Menthae*, Radix *Glycyrrhizae*	Harmonizing the stomach, regulating Qi, relieving pain, and curing epigastric distention, soreness, reflux and acid regurgitation	[21]
Hui Sheng Kou Fu Ye	Pericarpium *Zanthoxyli*, Lignum *Sappan*, Herba *Leonuri*, Flos *Carthami*, Radix *Angelicae Sinensis*, Rhizoma *Chuanxion*, *Hirudo*, Rhizoma *Sparganii*, Rhizoma *Anemones Raddeanae*	Removing blood stasis, and curing primary liver cancer, lung cancer	[21]
Fu Fang Zhi Zi Qi Wu Ji	Pericarpium *Zanthoxyli*, Radix *Sophorae Flavescentis*, Fructus *Gardeniae*, Radix *Arnebiae Seu Lithospermi*, Radix *Sanguisorbae*, *Borneolum Syntheticum*, Radix and Rhizoma *Rhei*, Rhizoma *Coptidis*, Flos *Sophorae*, Herba *Asari*	Clearing heat, detoxicating, stopping bleeding, relieving swelling and pain, and curing incising wounds, acne of the superficial skin	[21]
Bing Zhi Shang Tong Qi Wu Ding	Pericarpium *Zanthoxyli*, Radix and Rhizoma *Rhei*, Fructus *Gardeniae*, Radix *Rehmanniae*, Lignum *Dalbergiae Odoriferae*, Radix *Allii Tuberosi*, Semen *Serychni*, *Borneolum Syntheticum*, Semen *Persicae*, *Nodus Pini*, Rhizoma *Zingiberis*	Clearing heat, detoxicating, cooling the blood, promoting blood circulation, and curing bruises, swelling, and pain caused by extravasated blood, as well as burns	[21]
Zhi Tong An Cha Ji	Pericarpium *Zanthoxyli*, Radix *Sophorae Flavescentis*, Flos *Lonicerae*, Radix *Rumicis Nepalensis*, Fructus *Aurantii*, Flos *Sophorae*	Clearing heat and wetness, cooling blood and stopping blood, and curing perianalpruritic induced by heat-dampness retention	[21]
Li Fu Kang Xi Ji	Pericarpium *Zanthoxyli*, Radix *Sophorae Flavescentis*, Cortex *Phellodendri*, Fructus *Cnidii*, Cortex *Dictamni*, Rhizoma *Coptidis*, Fructus *Kochiae*, Radix *Isatidis*, Radix *Paeoniae Rubra*, Radix *Polygoni Multiflori*, Rhizoma *Smilacis Glabrae*	Clearing heat and wetness, relieving itching, and curing leucorrhea disease, pruritus vulvae, infusorial vulvitis, bacterial vaginopathy	[21]
Qing Bai Jie Shen Xi Ye	Pericarpium *Zanthoxyli*, Radix *Angelicae Sinensis*, Radix *Sophorae Flavescentis*, Rhizoma *Coptidis*, Cortex *Phellodendri*, Fructus *Cnidii*, Radix *Astragali*, Radix *Polygoni Multiflori*, Fructus *Kochiae*, Folium *Isatidis*, Radix *Paeoniae Rubra*	Clearing heat and wetness, detoxicating, relieving itching, and curing pruritus vulvae, vulvitis, bacterial vaginopathy	[21]
Qu Fu Er Xiang Shuan	Pericarpium *Zanthoxyli*, *Resina Draconis*, *Oblibanum*, Fructus *Cnidii*, *Alumen*, *Borax*, *Realgar*	Removing putrid tissues and promoting the growth of new tissue, as well as curing cervical erosion	[21]
Ku Shen An Shi Jin	Pericarpium *Zanthoxyli*, Radix *Sophorae Flavescentis*, Herba *Verbenae*, Herba *Taraxaci*, Fructus *Cnidii*, *Galla Chinensis*, Radix *Stemonae*, *Alumen*	Clearing heat and wetness, relieving itching, and curing pruritus vulvae in females, scrotal eczema in males	[21]
Ri Shu An Xi Ye	Pericarpium *Zanthoxyli*, Radix *Sophorae Flavescentis*, Herba *Verbenae*, Herba *Taraxaci*, Fructus *Cnidii*, *Galla Chinensis*, Radix *Stemonae*, *Alumen*	Clearing heat and dampness, detoxicating, relieving itching, and curing pruritus vulvae in females, scrotal eczema in males	[21]

**Table 2:** Alkaloids isolated from *Z. bungeanum* (**1**–**35**).

No.	Name	Part of Plant	Ref.
**1**	Hydroxy-α-sanshool	Pericarps	[8]
**2**	α-Sanshool	Pericarps	[34]
**3**	Hydroxy-β-sanshool	Pericarps	[8]
**4**	β-Sanshool	Pericarps	[34]
**5**	Hydroxy-γ-sanshool	Pericarps	[8]
**6**	γ-Sanshool	Pericarps	[8]
**7**	(2*E*,4*E*)-2′-Hydroxy-*N*-isobutyl-2,4-tetradecadienamide	Pericarps	[8]
**8**	(2*E*,4*E*, 8*Z*)-2′-Hydroxy-*N*-isobutyl-2,4,8-tetradecatrienamide	Pericarps	[8]
**9**	(2*E*,4*E*,8*Z*,10*E*,12*E*)-l′-Isopropenyl-*N*-(2′-bisobutenyl)-2,4,8,10,12-tetradecapentaenamide	Pericarps	[8]
**10**	(2*E*,4*E*,8*Z*,11*E*)-2′-Hydroxy-*N*-isobutyl-2,4,8,11-tetradecatetraenamide	Pericarps	[8]
**11**	(2*E*,7*E*,9*E*)-*N*-(2-Hydroxy-2-methylpropyl)-6-ethoxy-11-hydroxy-dodeca-2,7,9-trienamide	Pericarps	[26]
**12**	(2*E*,7*E*,9*E*)-*N*-(2-Hydroxy-2-methylpropyl)-11-ethoxy-6-hydroxy-dodeca-2,7,9-trienamide	Pericarps	[26]
**13**	(2*E*,6*E*,8*E*)-*N*-(2-Hydroxy-2-methylpropyl)-10-hydroxy-5-oxo-undeca-2,6,8-trienamide	Pericarps	[26]
**14**	(2*E*,4*E*,8*E*,10*E*,12*E*)-2′-Hydroxy-*N*-isobutyl-2,4,8,10,12-tetradecatetraenamide	Pericarps	[31]
**15**	(2*E*,4*E*,8*E*,10*E*,12*E*)-*N*-Isobutyl-2,4,8,10,12-tetradecapentaenamide	Pericarps	[34]
**16**	(2*E*,4*E*,8*Z*,11*Z*)-*N*-(2-Hydroxy-2-methylpropyl)-2,4,8,11-tetradeeatetraenamide	Pericarps	[35]
**17**	(2*E*,6*E*,8*E*)-*N*-(2-Hydroxy-2-methylpropyl)-10-oxo-2,6,8-decatrienamide	Pericarps	[35]
**18**	2′-Hydroxy-*N*-isobytyl-[*trans*-2,6,8,10] dodecatetraenamide	Pericarps	[34]
**19**	(6*RS*)-(2*E*,7*E*,9*E*)-6-Hydroxy-*N*-(2-hydroxy-2-methylpropyl)-11-oxo-2,7,9-dodecatrienamide	Pericarps	[36]
**20**	*N*-[2-(3,4-Dimethoxyphenyl)ethyl]-3-phenyl-acrylamide	Pericarps	[37]
**21**	Bugeanumamide A	Pericarps	[38]
**22**	(11*RS*)-(2*E*,7*E*,9*E*)-11-Hydroxy-*N*-(2-hydroxy-2-methylpropyl)-6-oxo-2,7,9-dodecatrienamide	Pericarps	[38]
**23**	(10*RS*,11*RS*)-(2*E*,6*Z*,8*E*)-10,11-Dihydroxy-*N*-(2-hydroxy-2-methylpropyl)-2,6,8-dodecatrienamide	Pericarps	[38]
**24**	(6*RS*,11*RS*)-(2*E*,7*E*,9*E*)-*N*-(2-Hydroxy-2-methylpropyl)-6,11-dioxo-2,7,9-dodecatrienamide	Pericarps	[38]
**25**	(2*E*,4*E*,9*E*,11*E*)-*N*-(2-Hydroxy-2-methypropyl)-8-hydroxy-13-oxo-2,4,9,11-tetradecatetraenamide	Pericarps	[38]
**26**	(2*E*,4*E*,9*E*,11*E*)-*N*-(Hydroxy-2-methypropyl)-8,13-dihydroxy-2,4,9,11-tetradecatetraenamide	Pericarps	[38]
**27**	(2*E*)-6,6-Dimethoxy-*N*-(2-hydroxy-2-methylpropyl)-2-hexenamide	Pericarps	[37]
**28**	Zanthobungeanine	Roots	[32]
**29**	Demethoxy chelerythrine	Roots	[32]
**30**	11-Demethoxy chelerythrine	Roots	[32]
**31**	l-*N*-Acetylanonanine	Roots	[32]
**32**	Arnothianamide	Roots	[32]
**33**	Skimmianine	Pericarps	[32]
**34**	Haplopine	Pericarps	[33]
**35**	Kokusaginine	Pericarps	[33]

Alkaloids isolated from *Z. bungeanum*.

**Table 3:** Terpenoids isolated from *Z. bungeanum* (**36**–**103**).

No.	Name	Part of Plant	Ref.
**36**	Linalool	Pericarps	[9]
**37**	Limonene	Pericarps	[9]
**38**	Geraniol	Pericarps	[9]
**30**	*p*-Mentha-1,3,8-triene	Pericarps	[9]
**40**	Citronellal	Pericarps	[9]
**41**	Isopulegol	Pericarps	[9]
**42**	Hotrienol	Pericarps	[9]
**43**	4-Terpinenyl acetate	Pericarps	[9]
**44**	*cis*-*p*-2-Menthen-1-ol	Pericarps	[9]
**45**	*cis*-*p*-Mentha-2,8-dien-1-ol	Pericarps	[9]
**46**	Citronellyl acetate	Pericarps	[9]
**47**	*trans*-*p*-Mentha-2,8-dienol	Pericarps	[9]
**48**	*p*-Mentha-1,8-dien-4-ol	Pericarps	[9]
**49**	Cryptone	Pericarps	[9]
**50**	*trans*-Piperitol	Pericarps	[9]
**51**	*cis*-Carveyl acetate	Pericarps	[9]
**52**	*p*-Menth-1-en-9-al	Pericarps	[9]
**53**	*trans*-Carveol	Pericarps	[9]
**54**	*p*-Mentha-1,8(10)-dien-9-ol	Pericarps	[9]
**55**	Isopiperitenone	Pericarps	[9]
**56**	*p*-1,8-Menthadienyl-7 acetate	Pericarps	[9]
**57**	2,3-Dehydro-1,8-cineole	Pericarps	[9]
**58**	*trans*-Sabinene hydrate	Pericarps	[9]
**59**	*trans*-Sabinene hydrate acetate	Pericarps	[9]
**60**	Pinocarvone	Pericarps	[9]
**61**	Bornyl acetate	Pericarps	[9]
**62**	Myrtenal	Pericarps	[9]
**63**	*trans*-Pinocarveol	Pericarps	[9]
**64**	Myrtenol	Pericarps	[9]
**65**	α-Cubebene	Pericarps	[9]
**66**	α-Bergamotene	Pericarps	[9]
**67**	Germacrene B	Pericarps	[9]
**68**	γ-Cadinene	Pericarps	[9]
**69**	α-Calacorene	Pericarps	[9]
**70**	β-Terpineol	Pericarps	[39]
**71**	α-Terpineol	Pericarps	[40]
**72**	α-Terpinene	Pericarps	[40]
**73**	p-Cymene	Pericarps	[40]
**74**	Neryl acetate	Pericarps	[9]
**75**	Geranyl acetate	Pericarps	[9]
**76**	Carvone	Pericarps	[9]
**77**	β-Thujone	Pericarps	[9]
**78**	β-Myrcene	pericarps	[9]
**79**	*cis*-Carveol	Pericarps	[9]
**80**	4-Isopropyl-l-methyl-2-cyclohexen-l-ol	Seeds	[9]
**81**	Linalyl anthranilate	Pericarps	[41]
**82**	Caryophyllene oxide	pericarps	[41]
**83**	Germacrene D	Pericarps	[41]
**84**	Nerol	Pericarps	[41]
**85**	Eucalyptol	Pericarps	[41]
**86**	24-en-Cycloartenone	Seeds	[42]
**87**	Camphene	Pericarps	[43]
**88**	β-Caryophyllene	Pericarps	[43]
**89**	α-Cadinol	Pericarps	[43]
**90**	β-Elemene	Pericarps	[43]
**91**	Myrcene	Pericarps	[43]
**92**	Carvacrol	Pericarps	[43]
**93**	(*E*)-β-Ocimene	Pericarps	[43]
**94**	(*Z*)-β-Ocimene	Pericarps	[43]
**95**	Sabinene	Pericarps	[43]
**96**	*α*-Terpinyl acetate	Pericarps	[43]
**97**	Piperitone	Pericarps	[43]
**98**	α-Thujene	Pericarps	[43]
**99**	β-Pinene	Pericarps	[44]
**100**	β-Phellandrene	Pericarps	[45]
**101**	γ-Terpinene	Pericarps	[45]
**102**	α-Pinene	pericarps	[45]
**103**	Terpinolene	Pericarps	[45]

Terpenoids isolated from *Z. bungeanum*.

**Table 4:** Flavonoids isolated from *Z. bungeanum* (**104**–**129**).

No.	Name	Part of Plant	Ref.
**104**	Rutin	Leaves	[10]
**105**	Syringetin-3-glucoside	Leaves	[10]
**106**	Isorhamnetin-3-glucoside	Leaves	[10]
**107**	Quercetin 3-arabinoside	Pericarps	[10]
**108**	3,5,7,3′,4′-Pentahydroxyflavone	Leaves	[23]
**109**	Quercetin 3-*O*-α-l-rhamnoside	Leaves	[23]
**110**	Quercetin 3-*O*-β-d-glucoside	Leaves	[23]
**111**	Trifolin	Leaves	[23]
**112**	Quercetin 3-*O*-β-d-galactoside	Leaves	[23]
**113**	Kaempferol 3-*O*-α-l-rhamnoside	Leaves	[23]
**114**	Isorhamnetin 3-*O*-α-l-rhamnoside	Leaves	[23]
**115**	Kaempferol-7-rhamnoside	Leaves	[10]
**116**	Apigenin-8-*C*-glucoside	Leaves	[10]
**117**	Apigenin-8-*C*-arabinoside	Leaves	[10]
**118**	Quercetin-3-rutinoside-7-rhamnoside	Leaves	[10]
**119**	Kaempferol-3-rutinoside	Leaves	[10]
**120**	Quercetin 3′,4′-dimethyl ether 7-glucoside	Pericarps	[46]
**121**	Tamarixetin 3,7-bis-glucoside	Pericarps	[46]
**122**	Isorhamnetin 7-glucoside	Pericarps	[46]
**123**	3,5,6-Trihydroxy-7,4′-dimethoxy flavone	Pericarps	[46]
**124**	5-Feruloyquinic acid	Leaves	[10]
**125**	Chlorogenic acid	Leaves	[10]
**126**	Sitosterol β-glucoside	Pericarps	[46]
**127**	l-sesamin	Pericarps	[46]
**128**	Quinic acid	Leaves	[10]
**129**	Epicatechin	Leaves	[10]

Flavonoids isolated from *Z. bungeanum*.

**Table 5:** Fatty acids isolated from *Z. bungeanum* (**130**–**139**).

No.	Name	Part of Plant	Ref.
**130**	Nonanoic acid	Pericarps	[49]
**131**	Tetradecanoic acid	Seeds	[11]
**132**	Pentadecanoic acid	Seeds	[11]
**133**	Hexadecanoic acid	Seeds	[11]
**134**	Stearic acid	Seeds	[11]
**135**	Eicosoic acid	Seeds	[11]
**136**	Oleic acid	Seeds	[11]
**137**	Palmitoleic acid	Seeds	[49]
**138**	Linolenic acid	Seeds	[48]
**139**	Linoleic acid	Seeds	[48]

Fatty acids isolated from *Z. bungeanum*.

**Table 6:** Other chemical compounds isolated from *Z. bungeanum* (**140**–**149**).

No.	Name	Part of Plant	Ref.
**140**	Rosefuran	Pericarps	[9]
**141**	Myrcene epoxide	Pericarps	[9]
**142**	Perillene	Pericarps	[9]
**143**	Vanillic acid-4-glucoside	Leaves	[10]
**144**	β-Sitosterol	Roots	[32]
**145**	Daucosterol	Seeds	[47]
**146**	Isoimperatorin	Seeds	[47]
**147**	Methyl-4-hydroxyphenylacrylate	Pericarps	[50]
**148**	7-Methoxycoumarin	Pericarps	[50]
**149**	Xanthoxylin	Pericarps	[50]

Other chemical compounds isolated from *Z. bungeanum.*

**Table 7:** Pharmacological effects of *Z. bungeanum*.

Pharmacological Effects	Detail	Extracts/Compounds	Minimal Active Concentration/Dose	In Vitro/In Vivo	Ref.
	Regulation on gastrointestinal smooth muscle	WEZB	4.0 and 12 mg/mL (i.g.)	in vivo	[51,52]
Effect on the digestive system	Anti-ulcer effects	Water extracts of *Z. bungeanum* (WEZB)	2.5, 5, and 10 g/kg (i.g.(intragastric administration), crude herb mass equivalent)	in vivo	[53]
	Anti-diarrhea effects	PEZB	3.0 and 6.0 mL/kg (i.g.)	in vivo	[53]
		WEZB	5 and 10 g/kg (i.g., crude herb mass equivalent)	in vivo	[53]
	Inhibiting contraction of isolated duodenal smooth muscle	EOZB	0.1 mg/mL	in vitro	[54]
	Inhibiting contraction of isolated colon smooth muscle	EOZB	0.4 g/L (i.g.)	in vivo	[55]
	Alleviating DSS-induced experimental colitis	WEZB	0.5,1.0, and 2.0 g/kg (i.g., for 14 days)	in vivo	[56]
	Accelerating defecation	Hydroxy-α-sanshool (HAS)	50 mg/kg (per os (p.o.), crude herb mass equivalent)	in vivo	[57]
	Improving blood flow of the colon	HAS	0.3 mg/kg	in vivo	[57]
	Improving release of ADM from intestinal epithelial cells	HAS	0.3, 10, and 30 μmol/L	in vitro	[57]
	Enhancing long distance contraction of the proximal colon	HAS	3, 10, and 30 μM	in vitro	[58]
Effect on the nervous system	Blocking nerve impulse	Essential oils of *Z. bungeanum* (EOZB) and WEZB	20%	in vitro	[59,60]
	Anti-depressive effects on behavioral despair models	PEZB	50 mg/kg (i.g.)	in vivo	[61]
	Reducing time of tail suspension	PEZB	50 mg/kg (i.g., for 21 days)	in vivo	[62]
	Upregulate NE and 5-HT	PEZB	50 mg/kg (i.g., for 21 days)	in vivo	[63]
	Anti-depressive effects in the unpredictable stress model and ovariectomized model	PEZB	50 mg/kg (i.g., for 21 days)	in vivo	[64,65]
	Shorten the escape latency in mice	HAS	5 mg/kg (p.o.)	in vitro	[66]
	Inhibiting Aβ-induced neuronal apoptosis and reducing neuronal toxicity	gx-50	5 μM	in vitro	[37]
	Enhancing the cross-platform times	gx-50	1 mg/kg(i.p., for 2 months)	in vivo	[37]
	Inhibiting cytokine release	gx-50	500 μM	in vitro	[67]
	Enhancing neurite outgrowth	*Z. bungeanum* (ZP)-amide A, ZP-amide B, ZP-amide C	20 μM	in vitro	[26]
Effect on the circulatory system	Reducing CHOL, TG, LDL, increasing HDL-C	Seed oil of *Z. bungeanum* (SOZB)	5, 10, and 20 mL/kg (i.g., for 4 weeks)	in vivo	[68]
	Reducing HBV, HLV, CHOL, TG and increasing HDL-C	SOZB	2.5 mL/kg (i.g., for 10 weeks)	in vivo	[69]
	Reducing TG, TC, LDL-C, MDA, and NO	SOZB	2.5, 5, and 10 g/kg (i.g., for 30 days)	in vivo	[70]
	Relaxing contracted aortic muscle	EOZB	2.0, 4.0, 6.0, 8.0, and 10.0 μL/mL	In vitro	[71]
	Increased the survival rate of mice subjected to collagen-adrenaline	(Alpha-linolenic acid) ALA	250 mg/kg (p.o., for 10 days)	in vivo	[72]
	Prolonged hemorrhage and coagulation time	ALA and its mixture	50, 100, and 250 mg/kg (p.o., for 10 days)	in vivo	[72]
	Decreased platelet aggregation	ALA and its mixture	70 and 175 mg/kg (p.o., for 10 days)	in vivo	[72]
Anti-inflammatory and analgesic effects	Inhibiting dimethylbenzene-induced ear oedema, carrageenan-induced rat paw oedema and acetic acid-induced torsion	WEZB	2.5, 5.0, and 10 g/kg (i.g., for 3 days, crude herb mass equivalent)	in vivo	[73]
		DEZB	1.5, 3.0, and 6.0 mL/kg (i.g.)	in vivo	[73]
	Inhibiting dimethylbenzene-induced oedema ear, acetic acid-induced pain	EOZB	0.05, 0.1, and 0.2 g/kg (i.g., for 14 days)	in vivo	[74]
	Inhibiting hot-plate-induced pain and dextran-40-induced itch–scratch responses	Alkaloids of *Z. bungeanum* (AZB)	118, 236, and 472 mg/kg (i.g., for 3 days)	in vivo	[75]
	Inhibiting NO production	MEZB	200 μM	in vitro	[76]
	Inhibiting iNOS mRNA expression	4-*O*-β-d-Glucopyranosyldihydroferulic acid	IC_50_ = 6.5 μg/mL	in vitro	[76]
	Analgesic effect on formalin test	EEZB, MEZB	40 μL, 5% (i.p.)	in vivo	[77]
	Relieving pain on tail-flick test	EEZB	20 μL, 5% (i.p.)	in vivo	[77]
	Inhibiting the excitability of A*δ* mechanosensory nociceptors	HAS	IC_50_ = 70 ± 7 μM	in vitro	[78]
	Inhibiting effects on nitric oxide (NO) production in LPS-stimulated RAW 264.7 macrophages	ZP-amide D, ZP-amide E, ZP-amide F and ZP-amide G	IC_50_ = 48.7 ± 0.32, 27.1 ± 1.15, 49.8 ± 0.38, and 39.4 ± 0.63 μM, respectively	in vitro	[38]
Antioxidant effect	Reducing MDA	WEZB	0.0195, 0.039, and 0.156 mg/mL	in vitro	[79]
	Scavenging DPPH radicals	FWEZBL, FEEZBL and FAEZBL	IC_50_ = 24, 17.5, and 7.6 μg/mL, respectively	in vitro	[80]
		SOZB	Not mentioned	in vitro	[11]
		EEZBL, EAEEZBL, AEEZBL and MEEZBL	IC_50_ = 40.75 ± 0.21, 13.20 ± 0.85, 18.55 ± 0.35 and 85.85 ± 2.19 μg/mL, respectively	in vitro	[23,81]
		Polysaccharide of *Z. bungeanum* (PZB)	EC_50_ = 0.021 mg/mL	in vitro	[82]
		Three fractions (<10 kDa, 10~30 kDa, and >30 kDa) of polypeptide of *Z. bungeanum* seeds kernel (PZBSK)	10 mg/mL	in vitro	[83]
		Quercetin, Quercitrin, Quercetin-3-*O*-β-d-glucoside, Hyperoside, Rutin and Isorhamnetin 3-*O*-α-l-rhamnoside	IC_50_ = 0.009 ± 0.001, 0.011 ± 0.001, 0.012 ± 0.001, 0.011 ± 0.001, 0.016 ± 0.001, and 0.028 ± 0.001 mM, respectively	in vitro	[23]
	Reducing ferric and ABTS^+^ radical	AEEZBL	615.88 ± 1.86 and 2147.83 ± 23.08 μmol equiv. Trolox/g, respectively	in vitro	[23,81]
	Reducing Fe^3+^	PZB	EC_50_ = 0.011 mg/mL	in vitro	[82]
	Reducing hydroxyl radical	PZB	EC_50_ = 0.008 mg/mL	in vitro	[82]
	Chelating Fe^2+^	PZB	EC_50_ = 0.056 mg/mL	in vitro	[82]
	Decreased hexanal content, TBARS value, and LOX	EEZBL	0.015%, 0.030%, and 0.045%, for 8 days	in vitro	[25]
	Increased catalase, superoxide dismutase, and glutathione peroxidase activities, decreased PV TBARS values	EEZBL	0.018%	in vitro	[84]
		Chlorogenic acid, Hyperoside and Quercitrin	0.01%	in vitro	[84]
	Increased cell growth rate of *E. coli*	Quercetin, Quercitrin, Quercetin-3-*O*-β-d-glucoside, Hyperoside, rutin and Isorhamnetin 3-*O*-*α*-l-Rhamnoside	Not mentioned	in vitro	[22]
	Inhibitory capacity on lipid peroxidation	Vitexin, Quercitrin, Afzelin, Trifolin	IC_50_ = 0.014 ± 0.001 0.013 ± 0.005, 0.065 ± 0.003, and 0.040 ± 0.001 mM, respectively	in vitro	[23]
Anti-tumor effect	Anti-tumor effects on H_22_	EOZB	4 mg/mL	in vitro	[85]
	Inhibitory effects on the growth of tumor in mice	EOZB	10, 25, 50, and 100 mg/kg	in vivo	[85]
	Anti-tumor effects on HeLa, A549, k562	EOZB	IC_50_ = 11.2 ± 0.2, 6.26 ± 0.05 and 1.37 ± 0.03 mg/mL, respectively	in vitro	[86,87]
	Anti-tumor effects on PC_12_	EOZB	0.5 mg/mL~2.0 mg/mL	in vitro	[86,87]
	Anti-proliferative effect towards HaCaT	EOZB	IC_50_ = 0.024% (*v*/*v*, for 48 h)	in vitro	[88]
	Inhibiting PC-3 cells, HEp-2 cells, Hela cells, MFC-7 cells	EOZB	IC_50_ = 0.04%, 0.021%, 0.03%, and 0.023%, respectively	in vitro	[88]
	Anti-proliferation effects against HepG2 cells	Sanshools	0~250 μg/mL	in vitro	[89,90]
	Inducting apoptosis activity against HepG2 cells	Sanshools	(0~200 μg/mL)	in vitro	[89,90]
	Inducing apoptosis and inhibiting cell growth in HepG2 cells	EEZB	1 μg/mL~8 μg/mL(for 48 h)	in vitro	[91]
	Inhibitory effects against *SW620* cell	Hyperoside	IC_50_ = 19.51 ± 4.95 μM for 96 h	in vitro	[92]
	Inhibiting proliferation of HaCaT cells	d-Limonene, Terpinen-4-ol and β-Myrcene	IC_50_ = 0.009%, 0.028%, 0.013% (*v*/*v*, for 48 h), respectively	in vitro	[88]
Anti-bacterial and anti-fungal effects	Decreased viable count of *S. aureus* and *E. coli*	WEZB	5 mg/mL (for 4 days, crude herb mass equivalent)	in vitro	[79]
	Inhibitory effects against *Alternariasolani*, *B. theobromae*, *F. oxysporum* f.sp. *cucumerinum*, *F. oxysporum* f.sp. *niverum*, *B. maydis*, *L. maculans*, *M. grisea*, *R. cerealis*, *R. solani*, *V. Pirina*, and *V. dahlia*	EOZB	IC_50_ = 0.44, 0.48, 0.43, 0.48, 0.24, 0.13, 0.28, 0.27, 0.24, 0.41, and 0.32 mg/mL, respectively	in vitro	[43]
	Inhibiting the growth of *R. solani* and *R. cerealis* mycelia	EOZB	IC_50_ = 0.95 and 1.22 mg/mL, respectively	in vitro	[43]
	Inhibitory effects towards *B. subtilis*, *Salmonella*, *S.aureus*, *B. cereus*, *E. coli*, *P. vulgaris*, *P. Crtinum Thom*, *A. flavus*, *A. niger*, *R. Nigricans*, and *S.cerevisiae*	EOZB	Minimum bacteriacidal (or fungicidal) (MIC/MFC) concentrations = 25, 6.25, 25, 12.5, 12.5, 12.5, 12.5, 12.5, 12.5, 25, and 12.5 mL/L, respectively	in vitro	[93]
	Inhibiting food-borne bacteria *S. aureus*, *B. subtilis*, *B. cereus*, *B. Laubach*, and *E. coli*	EOZB	MIC = 5.0, 1.25, 2.5, 1.25, and 2.5 mg/mL, respectively. MBC = 20, 2.5, 10, 5.0, and 5.0 mg/mL, respectively	in vitro	[44]
	Inhibitory effects against *F. sulphureum*	EOZB and *α*-Pinene	MIC = 6.25% and 12.50%	in vitro	[40]
	Reducing the lesion diameter of potato inoculated with *F. Sulphureum*	EOZB and α-Pinene	6.25% and 12.50%	in vivo	[40]
	Inhibitory activity against *B. cinerea*, *P. oryzae*, *P. piricola*, *G. Cingulate*, and *V. pyrina*	EEZBL	IC_50_ = 11.82 ± 1.15, 12.31 ± 0.45, 39.48 ± 2.25, 13.00 ± 1.34, and 33.22 ± 3.61 mg/mL, respectively	in vitro	[81]
		Chloroform fraction of EEZBL	IC_50_ = 9.39 ± 0.07, 4.18 ± 0.08, 10.89 ± 1.62, 0.83 ± 0.24, and 5.35 ± 0.34 mg/mL, respectively	in vitro	[81]
Insecticide effects	Repellent activity against ants	Piperitone, 4-Terpineol, and Linalool	Not mentioned	in vitro	[94]
	Anti-insect effects towards *T. castaneum*	Petroleum ether, Dichloromethane and Diethyl ether fraction of the EOZB	LD_50_ = 0.0713, 0.11699, and 0.12267 μL	in vitro	[95]
		MEZB	0.5, 1.0, and 1.5 mg/mL	in vitro	[96]
	Anti-insect activity against *aedes albopictus*	EOZB	15, 25, 35, and 45 μg/mL	in vitro	[96]
	Insecticidal effects against *L. serricorne* adults	EOZB obtained hydrodistillation and supercritical fluid CO_2_	LC_50_ = 3.99 and 12.54 μg/mL	in vitro	[41]
	Anti-insect activity against *L. serricorne*	Eucalyptol, Limonene, γ-Terpinene, Linalool, *α*-Terpineol and 4-Terpinenol	LC_50_ = 5.18, 14.07, 12.01, 18.04, 3.27, and 6.90 mg/L, respectively	in vitro	[41]
	Contact toxicity against *L. serricorne*	Eucalyptol, Limonene, γ-Terpinene, Linalool, α-Terpineol and 4-Terpinenol	LD_50_ = 15.58, 13.66, 14.19, 12.74, 11.99, and 8.62 μg/adult, respectively	in vitro	[41]
Other pharmacological effects	Relaxing subcutaneous muscles	Sanshools	Not mentioned	in vivo	[97]
	Alleviating rice-seedling injury	Sanshools	0.8 mg/mL	in vitro	[98]
	Prolonging the LPIA	Seeds of *Z. bungeanum* (SZB)	0.25, 0.5, and 1.0 g/kg	in vitro	[48]
	Reduce the cough number	SZB	0.25, 0.5, and 1.0 g/kg	in vitro	[48]
	Anti-fatigue and anti-anoxia ability	SZB	0.5, 1.0, and 2.0 g/kg (i.g.)	in vitro	[48]
	Enhancing the percutaneous absorption	EOZB	3%	in vitro	[45]
		Terpinen-4-ol,1,8-Cineole and Limonene	3%	in vitro	[45]
	Decrease serum TC and TG level	*n*-Butanol fraction of *Z. bungeanum*	50 mg/kg and 200 mg/kg (i.g., for 4 weeks)	in vivo	[99]
	Decrease TC, TG, FC level, apoB secretion, and increased apoA1	*n*-Butanol fraction of *Z. bungeanum*	0.05, 0.1, and 0.2 mg/mL	in vitro	[99]

Pharmacological effects of *Z. bungeanum*.

**Table 8:** Toxicities and side effects of *Z. bungeanum*.

Extracts/ Compounds	Animal/ Cell Line	Minimal Toxic Concentration/Dose	Toxic Effects	Ref.
WEZB	Mice	LD_50_ = 45 g/kg (i.g., crude herbs mass equal)	Death	[16]
WEZB	Mice	LD_50_ = 51.14 g/kg (i.g., crude herbs mass equal)	Death	[100]
WEZB	Mice	0.5, 1.0, 2.0 and 4.0 g/kg (i.g.)	Ballooning degeneration, cytoplasm rarefaction	[103]
EOZB	Mice	(LD_50_ = 2.27, 2.03, 4.64 and 5.32 g/kg of i.g., i.p., i.m., i.h., respectively	Death	[104]
EOZB	HaCaT cells and CCC-ESF-1 cells	IC_50_ = 2.435 mg/mL and 3.649 mg/mL, respectively	Inducing cell viability	[45]
WEZB	J774.1 cells	100, 200, and 400 μg/mL (for 18 h)	Non-toxic	[56]

Toxicities and side effects of *Z. bungeanum.*

## Abbreviations

AEEZBL	acetone fraction of the ethanol extraction of *Z. bungeanum* leaves
AZB	alkaloids of *Z. bungeanum*
AZBL	acetone fraction of *Z. bungeanum* leaves
CZBL	chloroform fraction of *Z. bungeanum* leaves
EAEEZBL	ethyl acetate fraction of the ethanol extraction of *Z. bungeanum* leaves
EAZBL	ethyl acetate fraction of *Z. bungeanum* leaves
EEZB	ethanol extracts of *Z. bungeanum*
EEZBL	ethanol extracts of *Z. bungeanum* leaves
EFZBL	ethanol fraction of *Z. bungeanum* leaves
EOZB	essential oils of *Z. bungeanum*
EZBL	extract of *Z. bungeanum* leaves
EZBP	extract of *Z. bungeanum* pericarps
FAEZBL	flavonoids of acetone extraction of *Z. bungeanum* leaves
FAZB	fatty acids of *Z. bungeanum*
FEEZBL	flavonoids of ethanol extraction of *Z. bungeanum* leaves
FWEZBL	flavonoids of water extraction of *Z. bungeanum* leaves
gx-50	(*E*)-*N*-[2-(3,4-dimethoxyphenyl)ethyl]-3-phenylacrylamide
HAS	hydroxyl-α-sanshool
MEEZBL	methanol fraction of the ethanol extraction of *Z. bungeanum* leaves
MEZB	methanol extracts of *Z. bungeanum*
MZBL	methanol fraction of *Z. bungeanum* leaves
PEEZB	petroleum ether extracts of *Z. bungeanum*
PEZB	polyphenol extracts of *Z. bungeanum*
PEZBL	petroleum ether fraction of *Z. bungeanum* leaves
PZB	polysaccharide of *Z. bungeanum*
PZBSK	polypeptide of *Z. bungeanum* seeds kernel
SOZB	seeds oil of *Z. bungeanum*
SZB	seeds of *Z. bungeanum*
WEZB	water extracts of *Z. bungeanum*
ZP-amide A	(2*E*,7*E*,9*E*)-*N*-(2-hydroxy-2-methylpropyl)-11-ethoxy-6-hydroxy-dodeca-2,7,9-trienamide
ZP-amide B	(10*RS*,11*SR*)-(2*E*,6*Z*,8*E*)-10,11-dihydroxy-*N*-(2-hydroxy-2-methylpropyl)-2,6,8-dodecatrienamide
ZP-amide C	(10*RS*,11*RS*)-(2*E*,6*Z*,8*E*)-10,11-dihydroxy-*N*-(2-hydroxy-2-methylpropyl)-2,6,8-dodecatrienamide
ZP-amide D	(2*E*,4*E*,9*E*,11*E*)-*N*-(2-hydroxy-2-methypropyl)-8-hydroxy-13-oxo-2,4,9,11-tetradecatetraenamide
ZP-amide E	tetrahydrobungeanool
ZP-amide F	(6*RS*)-(2*E*,7*E*,9*E*)-6-hydroxy-*N*-(2-hydroxy-2-methylpropyl)-11-oxo-2,7,9-dodecatrienamide
ZP-amide G	(2*E*,7*E*,9*E*)-*N*-(2-hydroxy-2-methylpropyl)-6,11-dioxo-2,7,9-dodecatrienamide
